# Development and Evaluation of an In Silico Dermal Absorption Model Relevant for Children

**DOI:** 10.3390/pharmaceutics14010172

**Published:** 2022-01-12

**Authors:** Yejin Esther Yun, Daniella Calderon-Nieva, Abdullah Hamadeh, Andrea N. Edginton

**Affiliations:** School of Pharmacy, University of Waterloo, Waterloo, ON N2G 1C5, Canada; y5yun@uwaterloo.ca (Y.E.Y.); dcalderonnieva@uwaterloo.ca (D.C.-N.); ahamadeh@uwaterloo.ca (A.H.)

**Keywords:** dermal absorption, physiologically based modeling, neonatal skin

## Abstract

The higher skin surface area to body weight ratio in children and the prematurity of skin in neonates may lead to higher chemical exposure as compared to adults. The objectives of this study were: (i) to provide a comprehensive review of the age-dependent anatomical and physiological changes in pediatric skin, and (ii) to construct and evaluate an age-dependent pediatric dermal absorption model. A comprehensive review was conducted to gather data quantifying the differences in the anatomy and physiology of child and adult skin. Maturation functions were developed for model parameters that were found to be age-dependent. A pediatric dermal absorption model was constructed by updating a MoBi implementation of the Dancik et al. 2013 skin permeation model with these maturation functions. Using a workflow for adult-to-child model extrapolation, the predictive performance of the model was evaluated by comparing its predicted rates of flux of diamorphine, phenobarbital and buprenorphine against experimental observations using neonatal skin. For diamorphine and phenobarbital, the model provided reasonable predictions. The ratios of predicted:observed flux in neonates for diamorphine ranged from 0.55 to 1.40. For phenobarbital, the ratios ranged from 0.93 to 1.26. For buprenorphine, the model showed acceptable predictive performance. Overall, the physiologically based pediatric dermal absorption model demonstrated satisfactory prediction accuracy. The prediction of dermal absorption in neonates using a model-based approach will be useful for both drug development and human health risk assessment.

## 1. Introduction

Mathematical in silico models of skin permeation simulate the dermal permeation and systemic exposure of a chemical through human skin. Given the limited availability of human and animal skin samples for permeability experiments and the differences between human and animal skin, in silico models can be used in lieu of experimental studies to estimate dermal exposure to chemicals and drugs and to predict systemic exposure under various dosing conditions and exposure scenarios [1]. This is especially valuable in pediatric patients where skin samples for in vitro studies are even more limited.

The mechanistic dermal absorption model by Dancik et al. [2] integrates a series of pharmacokinetic models, as previously described [3,4,5,6,7,8,9], that represent the penetration pathways of a chemical through skin. The components of the model were derived using data gathered from in vitro studies of chemical permeation in animal models and adult human skin samples [1]. The model also incorporates structural and physiological properties of adult human skin, which have been extensively described in detail [4,10,11,12,13,14,15]. The model can generate longitudinal estimates of the flux (e.g., µm/cm^2^/h) and accumulation (e.g., µm/cm^2^) of small molecule compounds in the various skin layers under both in vitro and in vivo conditions. In the in vivo context, the model can additionally generate estimates of the bioavailability of dermally absorbed chemicals.

Pediatric exposure to environmental chemicals is an important component of human health risk assessment. Although rare, cases of chemical poisoning through skin exposure have been reported in pediatric patients under 17 years of age [16]. This is especially concerning given the presence, in children’s bath products, of chemicals such as 1,4-dioxane and formaldehyde, which have been classified as carcinogens by the US Environmental Protection Agency (EPA) [17]. Moreover, the French Agency for Food, Environmental and Occupational Health and Safety has reported the identification of 60 hazardous chemicals in infant disposable diapers [18]. An increase in skin exposure to harmful chemicals may be a serious health risk in children, given the higher skin surface area to body weight ratio and the prematurity of skin in neonates [19]. Accounting for the anatomical and physiological changes in skin associated with age using the Dancik et al. model [2] may therefore help to guide the risk assessment of chemicals and pharmaceutical products in children.

Skin development and maturation begins in utero, and a full-term infant’s skin is histologically similar to adult skin, as it has a well-defined stratum corneum in addition to the other epidermal layers [20,21]. However, in vivo studies using confocal laser scanning microscopy in the last 20 years have shown that differences in skin anatomy and physiology do exist as a function of age following birth, which was not previously well-captured in light microscopy and chemically fixed skin samples in infants. Physiological and structural skin features that differ between infants and adults, which were identified from the analysis of non-invasive in vivo measurements, have been previously reviewed [22]. These variations in pediatric skin anatomy and physiology with respect to adults can induce differences in the dermal absorption of a given chemical between the two populations. As a result, in silico predictions generated by dermal models tailored to adult skin may fail to correctly predict exposure in the pediatric population. The objectives of this study were therefore: (i) to provide a comprehensive review of the anatomical and physiological changes associated with the skin of children and (ii) to construct and evaluate a pediatric dermal absorption model that accounts for skin maturation with age.

## 2. Materials and Methods

### 2.1. Dermal Absorption Modeling Preliminaries

**Figure 1:**
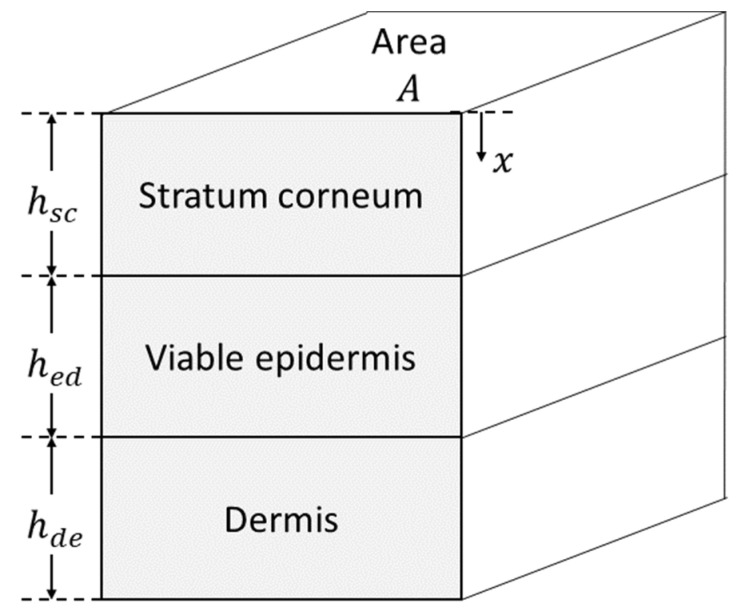
Structure of skin in the dermal absorption model.

The Dancik et al. [2] skin permeation model has previously been programmed into MoBi (Open Systems Pharmacology v.8.21) and is currently available on GitHub (https://github.com/Open-Systems-Pharmacology/Skin-permeation-model, accessed on 1 November 2021). The predictive accuracy of this model for the case of volatile vehicles was evaluated in Hamadeh et al. [23] with respect to in vitro skin permeation data reported in Hewitt et al. [24]. The model assumes that skin sections are composed of three stacked compartments that correspond to the stratum corneum (SC), the epidermis (ED) and the dermis (DE) (Figure 1), which have the respective thicknesses $h_{sc}$, $h_{ed},\;\mathrm{and}\;\;h_{de}$. The permeating compound is applied via a vehicle to the surface of the SC. The applied permeant is assumed to subsequently diffuse into the skin according to Fick’s law of diffusion [5] as detailed in Dancik et al. [2]. The permeant in each compartment can partition into sub-compartments that represent different phases within the skin sublayers, such as the lipid, protein or aqueous phases. The aggregate, layer-specific, diffusivity and partitioning processes in each skin layer are quantified by the diffusion coefficients ($D_{sc}$, $D_{ed}$, $D_{de}$) and the partitioning coefficients ($K_{sc/w}$, $K_{ed/w}$, $K_{de/w}$), respectively. These aggregate coefficients can be decomposed into diffusion and partition coefficients specific to each phase, as detailed in Dancik et al. [2]. These more fundamental coefficients can, furthermore, be decomposed into quantitative structure property relationships (QSPRs) that are functions of the physical/chemical properties of the permeant. The permeant concentration at depth x, as measured from the top of the SC, at time t, can be expressed as c(x,t). The complete partial differential equation model (PDE) describing permeant diffusion and clearance from the skin can be found in [2,25].

### 2.2. Physiological and Anatomical Changes in Skin as a Function of Age

A comprehensive literature review was conducted of the anatomical and physiological properties of skin that impact dermal absorption according to the Dancik et al. [2] model. Skin-specific parameters of this model include the stratum corneum thickness, the thickness of the viable epidermis, dermis thickness, stratum corneum hydration, lipid, and protein fraction of the stratum corneum, skin surface pH, corneocyte size and volume fraction of the stratum corneum, follicle size and density, albumin concentration in skin, and skin blood flow. Each parameter was searched on MEDLINE and EMBASE or PUBMED to identify articles that reported quantifiable data in healthy full-term infants and children up to 18 years of age (Tables S1–S6). The search strategy for each parameter is displayed in the Supplementary Materials Tables S7–S14. The results were limited to the English language and human studies. Child and adult estimates were obtained for the stratum corneum thickness, epidermis thickness, dermis thickness, and skin hydration.

### 2.3. Development of an Age-Dependent Dermal Absorption Model

#### 2.3.1. Development of Model Structure

We generalized the dermal absorption model in [2,25] to include the effect of subject postnatal age (Age). This model can be expressed using the following shorthand representation:

(1) $$\mathrm{PDE}\;\frac{\partial c}{\partial t}=f\left(t,c,\frac{\partial c}{\partial x},\frac{\partial^{2}c}{\partial x^{2}},P,P_{A}\left(Age\right)\right)$$

(2) $$\mathrm{Initial}\;\mathrm{conditions}\;c\left(0\right)=c_{0}\left(P,P_{A}\left(Age\right)\right)$$

(3) $$\mathrm{Boundary}\;\mathrm{conditions}\;h\left(c,\frac{\partial c}{\partial x},P,P_{A}\left(Age\right)\right)=0$$

(4) $$\mathrm{Model}\;\mathrm{outputs}\;y\left(t\right)=g\left(t,P,P_{A}\left(Age\right)\right)$$

The model represented by (1)–(4) is assumed to have the same structure as the model in [2,25]; however, we allowed for some of the parameters in the original model to vary with postnatal age. Model parameters that do not change with postnatal age were grouped into the parameter set P, while postnatal age-dependent parameters were grouped into the set $P_{A}\left(Age\right)$. The skin layer thicknesses ($h_{sc}$, $h_{ed},\;\;h_{de}$), permeant diffusivities ($D_{sc}$, $D_{ed}$, $D_{de}$) and partitioning coefficients ($K_{sc/w}$, $K_{ed/w}$, $K_{de/w}$) can be expressed as lump parameters that are functions of the parameters in the sets P and $P_{A}\left(Age\right)$.

For the purposes of this study, the model outputs y(t) were limited to estimates of two in vitro skin permeation test (IVPT) observations: (1) the permeant flux from the dermis into receptor fluid ($y_{J}\left(t\right)$), and (2) the permeant accumulation in receptor fluid ($y_{Q}\left(t\right)$). Assuming receptor fluid conditions that replicate permeant solubility and diffusivity in the dermis, these estimates can be derived from Fick’s law as models (5) and (6):

(5) $$y_{J}\left(t\right)=D_{de}{\frac{\partial c\left(x,t\right)}{\partial x}}_{|x=h_{sc}+h_{ed}+h_{de}\;}$$

(6) $$y_{Q}\left(t\right)=\int_{\tau =0}^{\tau =t}D_{de}{\frac{\partial c\left(x,\tau \right)}{\partial x}}_{|x=h_{sc}+h_{ed}+h_{de}\;}d\tau$$

#### 2.3.2. Maturation Models for Age-Dependent Model Parameters

Candidate models of postnatal age-dependence for each parameter within the set $P_{A}$ were developed as functions of postnatal age (in days) from birth to adulthood. Each such parameter was assumed to be expressible as a scaling with respect to a reference adult value $P_{adult}$ that depends on postnatal age (Age). The $i^{th}$. postnatal age-dependent parameter, $P_{A_{i}}\left(Age\right)$, was assumed, a priori, to have one of three candidate functional forms with respect to Age:

(7) $$\mathrm{Sigmoid}\;\mathrm{equation}\;\frac{P_{A_{i}}\left(Age\right)}{P_{adult}}=\frac{a\;\cdot \;Age}{b+Age}+c$$

(8) $$\mathrm{Hill}\;\mathrm{equation}\;\frac{P_{A_{i}}\left(Age\right)}{P_{adult}}=\frac{a\;\cdot \;Age^{n}}{b^{n}+Age^{n}}+c$$

(9) $$\mathrm{Polynomial}\;\mathrm{equation}\;\frac{P_{A_{i}}\left(Age\right)}{P_{adult}}=a\cdot \;Age^{n}+b\cdot Age^{m}+c$$

The nlstools R package (R version: 3.6.1, nlstools version: 1.0-2) [26,27] was used to fit each of the models (5)–(7) for each postnatal age-dependent parameter to the literature-sourced data collected through the literature review. To evaluate the test error rate of the models, leave-one-out cross validation (LOOCV) [28] was carried out. For each parameter, the functional form with the lowest LOOCV test error was selected as the final model.

For each $\frac{P_{A_{i}}\left(Age\right)}{P_{adult}}$ ratio used in model optimization, the values of $P_{A_{i}}\left(Age\right)$ and $P_{adult}$ were sourced, where possible, from the same study from the literature. Mean levels in adults of the SC, ED and DE thicknesses ($h_{sc}$, $h_{ed},\;\;h_{de})$ were collected, and the geometric mean of those mean values was calculated as a reference level in adults. When the adult level was not reported in the same study as the child level, a reference level in adults was used for $P_{adult}$. This reference level was estimated as the mean of all adult values collected in the literature.

### 2.4. Age-Dependent Dermal Absorption Model Optimization and Evaluation

The MoBi dermal absorption model was updated with the optimized maturation functions $P_{A}\left(Age\right)$ to form an integrated, postnatal age-dependent, dermal absorption model. The ability of the integrated model to capture changes in dermal absorption across postnatal age was evaluated using literature-sourced data on the skin permeation by three compounds: buprenorphine, diamorphine, and phenobarbital. These three compounds were selected based on the availability of experimental in vitro skin penetration data in adults and infants within the same study [29,30,31]. To assess the predictive performance of the model, the difference between observed and predicted flux values (fold error) was calculated by using Equation (10).

(10) $$fold\;error=\frac{Predicted\;flux\;\left(y_{J}\right)}{Observed\;flux}$$

#### 2.4.1. Dermal Absorption Model Sensitivity Analysis and Parameter Uncertainty

A local sensitivity analysis was conducted on the adult (Age = 30 years) models (1)–(4) for each of the three compounds to identify the uncertain model parameters that strongly impact estimates of the outputs $y_{J}$ and $y_{Q}$. For each of buprenorphine, diamorphine, and phenobarbital, these sensitivities were evaluated after updating the model with the compound’s corresponding parameters in Table 1 and Table 2. The uncertain model parameters to which the outputs $y_{J}$ and $y_{Q}$ are sensitive were classified into two sets: those that vary with postnatal age (denoted $P_{A}^{*}$) and those that are independent of postnatal age (denoted $P^{*}$).

A probability distribution for parameters $P_{A}^{*}$ for adults was obtained from the literature. Parameters $P_{A}^{*}$ for different ages were assumed to be distributed according to a corresponding probability distribution that is conditional on postnatal age, $p(P_{A}^{*}|Age)$. A sample from this conditional distribution is obtained, first, by sampling the adult distribution for parameters $P_{A}^{*}$, and then scaling the sample according to the optimized maturation model.

#### 2.4.2. Model Optimization and Evaluation

For each compound, the model evaluation consisted of the following steps:

S1. The models (1)–(4) were updated with the compound-specific parameters from Table 1 and age-dependent parameters corresponding to adult skin (in which parameter Age=30 years) gathered from the PubChem database [32].
S2. The uncertain age-independent parameters $P^{*}$, to which the model outputs (5) and (6) are sensitive, were estimated by fitting outputs $y_{J}$ and $y_{Q}$ from the adult model generated in step S1 to the observed adult flux and receptor fluid accumulation in Table 2. Model fitting was performed via the Monte Carlo parameter identification algorithm in MoBi (Open Systems Pharmacology v.9.1), initiated from 10 randomly selected initial values.
S3. Infant skin permeation by the compound was simulated assuming the experimental conditions and skin ages in Table 3 using the optimized parameter values $P^{*}$ obtained in step S2. A total of 100 simulations of the model were run for each infant skin Age on which the compound was experimentally tested. Each such simulation was run after updating the model with a new sample from the distribution of age-dependent parameters $p(P_{A}^{*}|Age)$. For each experiment, the mean and 95% confidence intervals of the simulated permeant flux $y_{J}$ were evaluated and compared with the corresponding observed flux in Table 3.

Neonates who were born before a gestational age (GA) of 37 weeks were considered to be preterm, whereas infants who were born after 37 weeks (i.e., GA ≥ 37 weeks) were classified as full-term [33]. The observed data for full-term and late preterm neonates with a gestational age from 35 to 40 weeks were included in this study. The review of skin anatomy and physiology did not focus on pre-term neonates, and this evaluation was for preliminary assessment only.

### 2.5. Identification of Critical Input Parameter

To assess which parameters were both important to the outcome of flux and had an importance that differed between adults and children, a post hoc sensitivity analysis was performed. The age of adults was set to 30 years and the age of children was set to the same age in the corresponding studies. Parameters that were differentially sensitive with respect to age were identified by calculating the absolute difference in sensitivity coefficients between children and adults. If the difference was equal to or greater than 15% (Equation (11)), the parameter was considered to be age-sensitive.

(11) |sensitivity coefficient in children−sensitivity coefficient in adults| ≥15%

## 3. Results

### 3.1. Physiological and Anatomical Changes in Skin as a Function of Age

#### 3.1.1. Differences in Stratum Corneum Thickness

The geometrical properties of the stratum corneum (SC) are critical parameters used to determine the steady state permeation, lag time, and the flux of a substance transiting intercellularly through the SC [34]. Thus, studies investigating SC thickness in children were reviewed and quantifiable data were collected to determine whether SC thickness changed as a function of age.

A total of 43 relevant articles were identified in PUBMED, and 17 were identified as containing child-specific epidermis thickness data compared to adult epidermis thickness data (Supplementary Tables S1 and S7). It is important to note that the epidermis consists of the SC and the viable epidermis. The viable epidermis is distinct from the SC, as it contains nucleated keratinocytes, melanocytes, Langerhans cells, and Merkel cells [35]. Some investigations of epidermis thickness in children include SC thickness and identify it as the supra-papillary epidermis or epidermis thickness [36,37,38]. Of the 17 articles, only eight specifically measured SC thickness in children compared to adults. The body areas in which SC thickness was most often measured in children were the forearms, upper arm, and abdomen [21,36,37,38,39,40], although data from other body areas such as the buttocks and thighs have also been collected [36,37,38,40]. Measurements of histological skin samples ex vivo and confocal microscopy in vivo were the most common methods used to measure skin thickness.

Earlier studies measuring SC thickness using histological methods did not identify a significant distinct difference in the thickness of the SC between infants and adults [21,39,41,42]. This was unlike in vivo SC measurements using confocal microscopy and confocal Raman spectroscopy, which revealed that infant SC is thinner than adult SC [36,37,43]. Stamatas et al. [36] found that the SC thickness of lower thigh skin from 20 infants from 3 months to 2 years old was on average 30% thinner than adults (7.3 ± 1.1 µm versus 10.5 ± 2.1 µm). Similarly, Liu et al. [37] also recorded that 52 infants and children of the same age range had a 34% thinner SC in the lower thigh compared to adults. In the same study, SC thickness measurements at the upper inner arm also revealed a thinner SC in children compared to adults, although the magnitude of the difference was smaller, at only 18% thinner (5.3 ± 1.4 µm vs 7.9 ± 1.8 µm) [37].

Another investigation by Walters et al. [43] aimed to more closely identify the relationship between SC thickness and age by grouping infants and children 3 months old to 5 years old into different age bins. The SC thickness of the upper inner arm and dorsal forearm increased from 8 µm at 3 months of age to 14 µm at 4 years of age, at which point it became similar to adult (25–40 years old, average: 32 years) SC thickness, which ranged from 13 to 14 µm in this study. The SC thickness of dorsal forearm and inner arms were also similar in thickness in this study. Only one study was retrieved that measured SC thickness in neonates and infants less than 3 months of age [38]. This study pooled SC measurements of neonates aged 4–7 days old and compared this thickness to measurements taken at 1, 3 and 6 months after birth in the same infants. Measurements were taken from the buttock, thigh, and forearm skin. Unlike the previous studies, Miyauchi et al. [38] found that the SC was thicker at 4–7 days of age compared to 3 months of age at all three measured sites.

**Figure 2:**
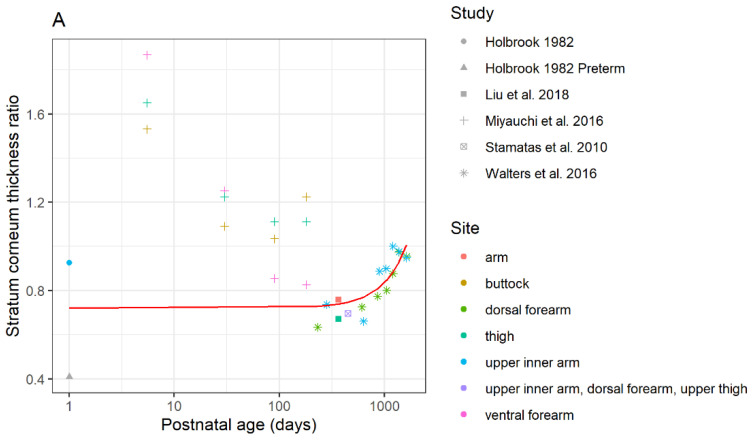

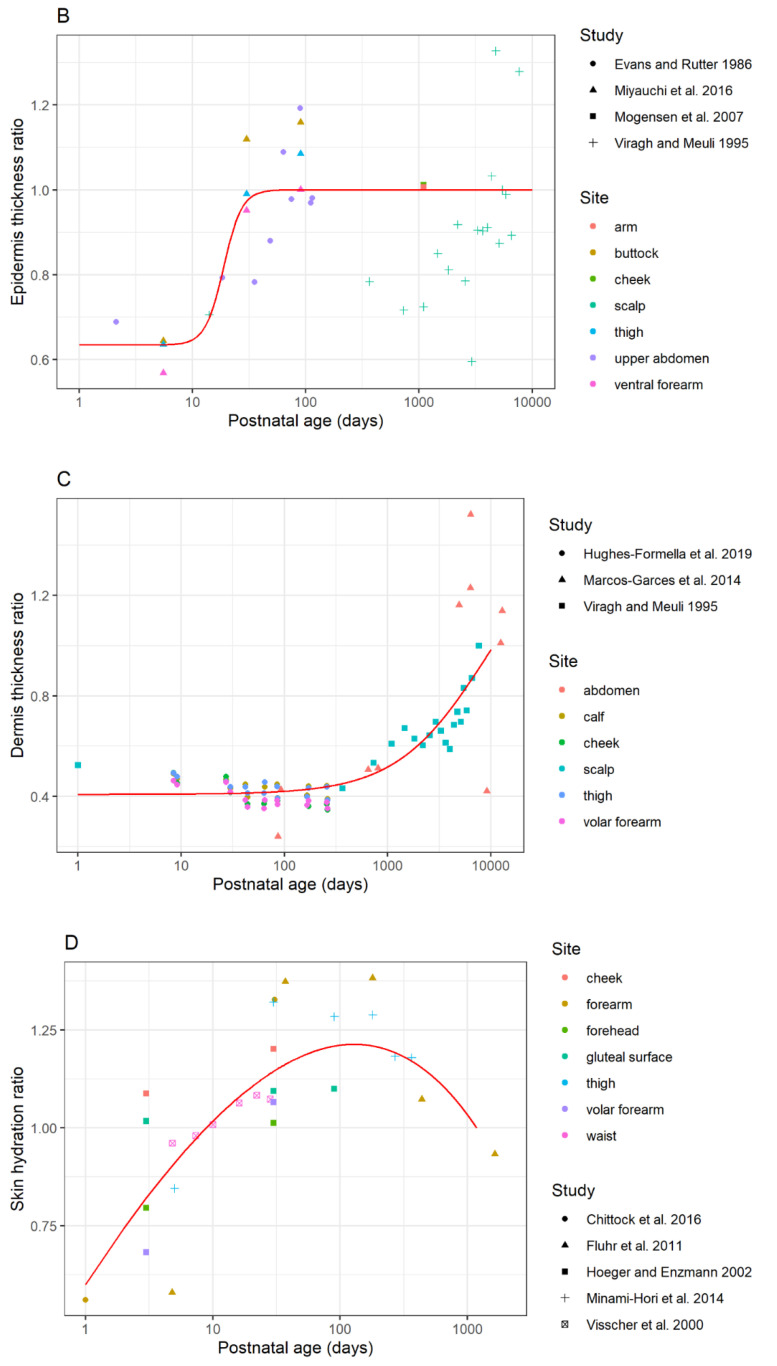
Maturation ratios vs. age profiles of (**A**) stratum corneum thickness (SC maturation model 2), (**B**) epidermis thickness, (**C**) dermis thickness, and (**D**) skin hydration. The model structures and coefficients are listed in Table 4. For the stratum corneum thickness model, Miyauchi 2016 data and Holbrook 1982 preterm data were not included in the development of SC maturation model 2.

The ratio of child SC thickness to adult SC thickness was plotted as a function of postnatal age (Figure 2A). Overall, SC thickness approaches adult values at around 4 years of age.

#### 3.1.2. Differences in the Thickness of the Viable Epidermis between Children and Adults

After diffusion through the SC, a chemical next permeates through the viable epidermis. Like SC thickness, the thickness of the viable dermis is important for calculating diffusion and permeability rates. A total of nine publications investigating epidermal thickness in children were identified (Supplementary Tables S2 and S8).

Measurements of abdominal viable epidermis thickness were first recorded by Evans and Rutter (1986). They measured the viable epidermal thickness of post-mortem infant skin samples ex vivo and identified that it increased linearly with postnatal age up to 16 weeks of life [21]. Moreover, they also recorded that the distinct undulating nature of the epidermis develops after birth and becomes more distinct with age. Similar findings were recorded by de Viragh et al. [44] a few years later in scalp skin isolated from biopsy specimens [44]. However, a distinction between maximum epidermis thickness and minimal epidermis thickness was identified. The maximum epidermis was defined as the distance from the start of the viable epidermis to the most prominent projection of the collagen fibers, which identifies the border between the epidermal and dermis skin layers. The maximum epidermis thickness increased with age, unlike the minimal epidermis thickness, which did not vary. This was similar to the study by Evans et al. (1986) [21], which identified an increase in the undulating nature of the epidermis.

Similarly, more recent studies measuring epidermal thickness in vivo also concluded that infants have a thinner epidermis than adults. Stamatas et al. [36] and Liu et al. [37], both found that pooled epidermis thickness values of thigh skin in infants aged 3–24 months were 20% and 8% lower, respectively, than adult values. Liu et al. [37] also measured a 22% thinner inner arm epidermis in children compared to adults. The changes in epidermis thickness in neonates was also more closely identified by Miyauchi et al. [38], where epidermal thickness was measured in four day old infants until they were 6 months of age. Given the undulating nature of the epidermis, two thickness values were measured, which corresponded with the top of the dermal papillae and the bottom of the rete ridges (i.e., bottom of dermal papillae). The epidermis thickness increased with age until one month of age, where it reached a thickness of 25 and 58 µm in minimal and maximal epidermis thickness, respectively [38]. At this time point, the maximal epidermis thickness was similar to adult maximal epidermis thickness (60 µm) [38]. A final study measuring epidermal thickness in children aged six months to three years of age also concluded that epidermal thickness is thinner in children compared to adults, but did not show the data for this [45].

The viable epidermis in children was thinner than the adult epidermis. Since the data collected from Evans et al. [21], de Viragh et al. [44], Miyauchi et al. [38], and Mogensen et al. [45] included thickness values stratified by age group, the ratio of child epidermis thickness to adult epidermis thickness was plotted as a function of postnatal age (Figure 2B). In terms of de Viragh et al. [44], as the minimum and maximum values were reported, the averages of the minimum and the maximum values were used. These data outline that the epidermis thickness in the first week of life is thinner than in adults, and remains relatively similar until 10 days postnatal age, at which point the epidermal thickness increases rapidly until four months of age, where it reaches adult values.

#### 3.1.3. Differences in the Dermal Thickness between Children and Adults

The dermis layer of the skin is the thickest layer, and although it contributes to a significant amount of variability for in vitro experiments [46], this layer is important when predicting systemic drug delivery through the transdermal route [47]. As such, differences in dermis thickness ($h_{de})$ between children and adults were investigated. A total of four relevant articles were identified as having quantifiable data of dermis thickness values in children (Supplementary Tables S3 and S9). It is important to note that of the four articles, one was grey literature and the data were not extracted [48].

As with the epidermal layer, the dermis layer also has an undulating structure because of the dermal papillae. Additionally, it is made up of two layers: the papillary and reticular dermis. As such, thickness values were measured in a different way in each publication. Dermis thickness values from children aged one week to three years old were attained [44,49], in addition to children aged 18 years [50]. Scalp dermis thickness data sourced from De Viragh et al. [44] identified an increase in maximal dermis thickness as a function of age from 1125 µm at 2 weeks old to 1500 µm at 21 years of age. The minimal dermis thickness in this study also increased from 850 µm at 2 weeks old to 2200 µm at 21 years of age. Marcos et al. [50] also found a similar trend in skin samples obtained from 5-month-old infants up to 95 years of age. They found a thickness of 1603.88 µm at birth and 3236.18 µm in adults at 50 years of age. Finally, Hughes et al. [49] more closely identified the relationship between age and dermis thickness in infants that were 1 week old up to 3 years old. However, the bounds of the dermis that were measured to gather thickness values were not clear in this study. They found dermal thickness in the forearm to be highest at 1 week of age (1200 µm), which decreased to 1100 µm at 4 weeks of age and then was similar from 6 to 36 months of age at a thickness of 1050 µm.

The ratio of child dermis thickness to adult dermis thickness measured by de Viragh et al. [44], Marcos et al. [50], and Hughes et al. [49] was plotted as a function of postnatal age (Figure 2C). In terms of de Viragh et al. [44], as the minimum and maximum values were reported, the average of the minimum and the maximum values were used. The dermis thickness in children does not change and remains at around 40% of adult thickness until around 2 years of age (730 days postnatal), where the dermis thickness increases rapidly into adulthood and continues to increase until 27 years of age.

#### 3.1.4. Difference in Skin Hydration of Child and Adult Epidermal Barrier

The level of hydration of the skin and stratum corneum plays a role in permeability and chemical penetration. An increase in water content results in an increase in skin permeability, since the stratum corneum can act as a reservoir to promote percutaneous absorption [8,51]. Therefore, differences in surface skin hydration as a function of age in children were investigated. Skin hydration in the stratum corneum can be measured indirectly using a corneometer. The corneometer measures skin capacitance, which is related to the dielectric properties of the skin and is proportional to the water content in the skin [52]. A total of 16 publications measured surface skin hydration indirectly via skin capacitance (Supplementary Tables S4 and S10).

Several investigations have identified that newborns in the first 2 weeks of life have lower skin hydration than adults [53,54,55,56,57,58,59,60,61]. Chittock et al. [53] found that infants < 72 h old had skin capacitance of 17.66 ± 4.55 relative capacitance units (RCU), which was lower than adults at 31.47 ± 6.9 RCU. Similar trends were identified by Bartels et al. [55]. Additionally, a study by Yosipovitch et al. [61] also suggests that skin hydration begins significantly increasing in the first 24 hours of life [61]. As the neonate grows, skin hydration increases rapidly. The study by Bartels et al. [55] found that the highest increase in skin capacitance was by 7 arbitrary units (AU) in the abdomen from 2–7 days of age. Moreover, Visscher et al. [60] found that skin hydration continues to increase until 2 weeks of life then plateaus. However, several other investigations have found that the rapid increase in skin hydration in infants continues until approximately one month of age [55,57,58,59].

At the 1 month mark, the skin capacitance in the infant is higher than adults [58]. Visscher et al. [60] and Fluhr et al. [54] also identified similar trends. The study by Fluhr et al. (2012) [54] suggests that the hydration remains high above adult values until 6 months of age (41.5 AU) and decreases to adult values (30 AU) in the first 1–2 years of life. At 6 months to 1 year of age, several investigators have found that skin hydration in children is not significantly different than in adults [54,62,63].

The change in skin hydration as a function of postnatal age is shown in Figure 2D. Skin capacitance values from Chittock et al. [53], Fluhr et al. [54], Hoeger and Enzmann [57], Minami-Hori et al. [58], and Visscher et al. [60] were used to calculate a ratio of children’s skin hydration to adult skin hydration. These ratios are plotted as a function of postnatal age. As previously described, skin hydration increases until 1 month of age, then decreases to adult values.

#### 3.1.5. Differences in the Corneocyte Volume Fraction

The corneocyte phase of the SC is involved in model calculations that determine partition coefficients and saturation concentration of a substance in the SC [2]. The literature search identified four relevant articles related to differences in corneocyte size, shape, volume in the stratum corneum in children compared to adults (Supplementary Tables S5 and S11). Changes in cell density, cluster formation, cell shape, thickness, and adhesion in corneocytes of the stratum corneum exist in infants and children until 5 years of age, with the most drastic changes occurring during the first two years of life [2,3]. Corneocytes in the stratum corneum of infants from 6 to 24 months old were smaller than adult corneocytes, which was attributed to a higher proliferation rate of corneocytes in infants [5]. During infancy and into adulthood, corneocytes became larger and flatter and assumed a greater surface area, which was correlated with a decrease in proliferation rate [2,3,5]. Since the relationship between these data and the effect on the volume of corneocytes in the SC are unknown, the corneocyte phase volume fraction in children was kept the same as in adults in the model.

#### 3.1.6. Differences in the Lipid/Protein Ratio

Since the lipid contents in the SC, viable epidermis, and dermis affect the permeability of a substance through the corresponding layers, quantitative data regarding differences in lipid mass or volume between children and adults were investigated. Of the nine relevant articles identified looking at lipid composition in children, only two conference abstract articles from the same research group specifically measured whole lipid contents in infant skin compared to adults [64,65] (Supplementary Table S12). Stamatas et al. [64,65] measured the lipid content in the SC of the volar forearm of infants aged 3–24 months and their respective mothers. Similar amounts of urea, lipids (cholesterol and ceramides), and keratin (protein) were found in infants and adults. As a result, the same parameter values were used in adults and child simulations for the following parameters in the model: the protein phase volume fraction of the stratum corneum, the mass of proteins in relationship to the dry weight of the SC, and the mass of lipids in relation to the dry weight of the SC.

#### 3.1.7. Differences in Albumin Concentration

The albumin content in the skin affects chemical or drug protein binding in the skin and therefore unbound and bound concentrations within the dermis [2]. There are limited data regarding albumin content in full term infant skin, and a search only revealed one article that quantified albumin content in newborn skin [66] (Supplementary Table S14). From this article, it was evident that albumin concentration in premature infants is greater than adults and full-term newborns. However, the albumin content in adults and newborn skin were similar, both within 2.5–5 ng/µg of protein. As such, the parameter value of the fraction of aqueous phase accessible to albumin was kept the same between children and adults.

#### 3.1.8. Differences in Skin Blood Flow in Children

Skin blood flow is an important limiting parameter that helps to predict systemic drug clearance from the skin in vivo. In the Dancik et al. model [2], capillary clearance can be used to predict systemic clearance using the capillary surface area and estimated blood flow limited clearance. The modeling of dermal capillary clearance was reviewed by Kretsos and Kasting (2004) [67], who described several parameters such as geometry, vessel size, and surface area that affect capillary clearance. They also proposed a new microscopic model for the dermal capillary clearance process based on the physiologic capillary structure [68]. In infants, the microvascular structure is disorganized after birth and matures over the first 4–5 weeks post birth, when the papillary loops are seen as in adult skin [42]. More recently, Miyauchi et al. [38] also observed capillary loop formation in infants at 1 to 3 months of age. The relationship between vessel geometry and blood flow is complex, and several models are available with an aim to capture capillary transport [67]. An early study by Poschl et al. [69] identified that the skin blood flow in full-term and preterm neonates changes in the first week of life. In full-term neonates, the blood flow oscillations reached the lower range of the adult value within the 4 to 5 days of life [69]. The relationship between skin blood blow changes and microvessel structural changes in infant skin are not known and need to be further studied for future model development. Skin blood flow is not included in the Dancik et al. model [2]; however, the maturation of skin blood flow data will be useful in the in vivo prediction of dermal absorption.

#### 3.1.9. Differences in Surface Skin pH and Follicle Density/Size of Children and Adults

The skin pH is a crucial element of skin barrier function as it affects enzymatic activity in the skin and lipid processing [70]. Although surface skin pH is not an input parameter in the Dancik et al. model [2], the pH of newborn skin is near neutral, unlike in adults [71]. The differences between adult and child skin pH have been extensively and recently reviewed [70,71]. In short, skin surface pH in infants immediately after birth is higher and less acidic around 6.5 [60] than the pH in adult skin [70], which ranges from 4–6 [72]. The pH then decreases within 7–14 days and can normalize by 6 months [73]. Moreover, it appears similar to adults [62,73] in later infancy. While skin pH is not currently in the model, this review provides information that may be used in future.

Similarly, the transfollicular shunt route is another parameter not currently included in the Dancik model [2]. Given the importance of the transfollicular shunt route of drug permeation through the skin, differences in follicle size or density between children and adults were also investigated for future model development. The literature search on EMBASE and MEDLINE identified two articles with quantifiable data regarding follicle density, length, or diameter (Supplementary Tables S6 and S13). Marchini et al. [74] identified that the number of visible hair structures per mm^2^ in infants 1–2 days of age was approximately 10 times greater than in adults. Additionally, a grey literature source suggested that there may also be a relationship between hair follicle dimensions and age [75].

### 3.2. Development of a Dermal Absorption Model (Age-Dependent)

#### Maturation Models for Age-Dependent Model Parameters

**Figure A1:**
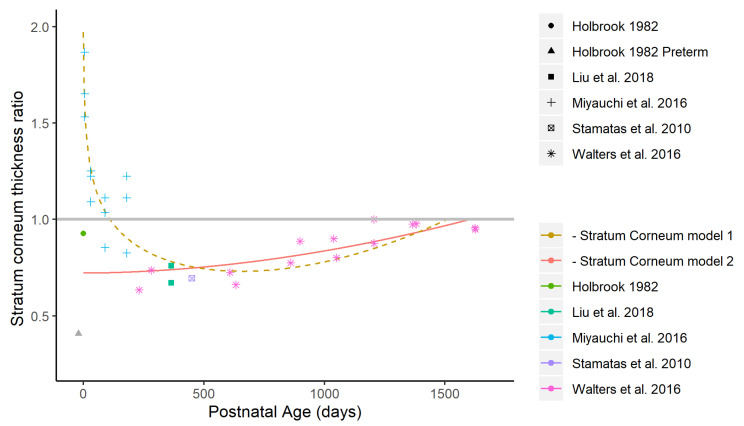
Alternative maturation model for stratum corneum thickness.

Based on the pediatric dermal data collated from literature, a predictive maturation model was developed for the stratum corneum thickness, epidermis thickness, dermis thickness, and stratum corneum hydration. Among the three tested models for each parameter, the model that resulted in the lowest test error value was selected as a final model. The final model equations and coefficients are listed in Table 4. For the SC thickness, a preliminary maturation (*SC Maturation Model 1* in Table 4) was constructed based on a dataset that includes measurements reported by Miyauchi et al., 2016 [38]. However, these data report SC thickness values in neonates that exceed values reported in adults (Appendix A Figure A1), in contradiction to previous literature findings. An alternate model, *SC Maturation Model 2* in Table 4, was therefore developed based on a dataset that excludes measurements from Miyauchi et al., 2016 [38]. The alternative model (*SC Maturation Model 2)* was chosen as the final model.

### 3.3. Age-Dependent Dermal Absorption Model Optimization and Evaluation

#### 3.3.1. Dermal Absorption Model Sensitivity Analysis and Parameter Uncertainty

**Figure 3:**
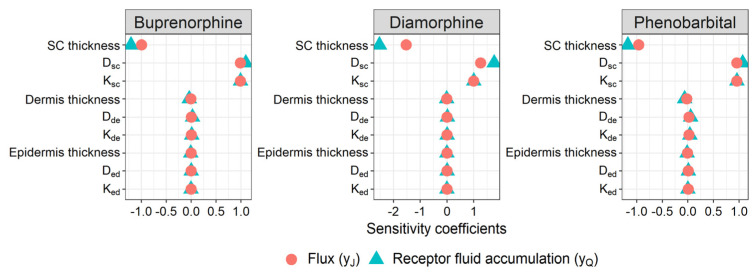
Local sensitivity analysis of the outputs $y_{J}$ and $y_{Q}$ of the dermal absorption models (1)–(4).

Local sensitivity analysis was conducted on the dermal absorption models (1)–(4) for adults for each of buprenorphine, diamorphine, and phenobarbital. This analysis assessed the impact on model outputs $y_{J}$ and $y_{Q}$ of local changes in the diffusion coefficients ($D_{sc}$, $D_{ed}$, $D_{de}$), partition coefficients ($K_{sc/w}$, $K_{ed/w}$, $K_{de/w}$) and skin layer thicknesses ($h_{sc}$, $h_{ed},\;\;h_{de}$). The results of the sensitivity analysis are shown in Figure 3. For all three compounds, sensitivity was highest with respect to the stratum corneum parameters ($D_{sc}$, $K_{sc/w}$, $h_{sc}$).

The SC diffusivity and partitioning coefficients, $D_{\mathrm{sc}}$ and $K_{\mathrm{sc}/w}$, are functions of three uncertain quantitative structure property relationships (QSPR): (1) the permeant trans-lipid bilayer permeability, denoted as $\log_{10}k_{\mathrm{trans}}$ [76]; (2) the permeant’s SC lipid phase/water partition coefficient, $K_{\mathrm{lip}/w}$ [77]; and (3) the permeant’s SC protein phase/water partition coefficient, ${\mathrm{PC}}_{\mathrm{pro}/w}$ [78]. Nominal values and uncertainties in these QSPRs are summarized in Table 5. These three parameters were taken to be the set $P^{*}$ of uncertain, age-independent model parameters.

The SC thickness, $h_{\mathrm{sc}}$, constitutes the set $P_{A}^{*}$ of age-dependent, uncertain parameters of the model. This quantity varies with the degree of SC hydration [2]. In the case of in vitro diffusion experiments, the SC is assumed to be fully hydrated, with a nominal thickness of 43 µm (Nitsche et al., 2006 [78]). The uncertainty in this model parameter for the case of adult skin, under in vitro (hydrated) conditions, was derived from literature-sourced measurements of the thickness of the partially hydrated SC at various anatomical sites, which are summarized in Table 6. First, the coefficient of variation in these measurements was estimated, under the assumption that they are log-normally distributed, to be 0.43. The fully hydrated SC thickness was similarly assumed to be log-normally distributed with a mean of 43 µm and a coefficient of variation equal to that of the partially hydrated SC measurements. From these estimates, the distribution in the fully hydrated $h_{\mathrm{sc}}$ for adults was approximated by $\mathrm{Lognormal}\;\left(\mu =3.68,{\;\sigma }^{2}=0.17\right)$, which is taken to be the distribution $p\left(P_{A}^{*}|\mathrm{Age}\right)$ for Age values representing adults.

#### 3.3.2. Model Optimization and Evaluation

**Figure 4:**
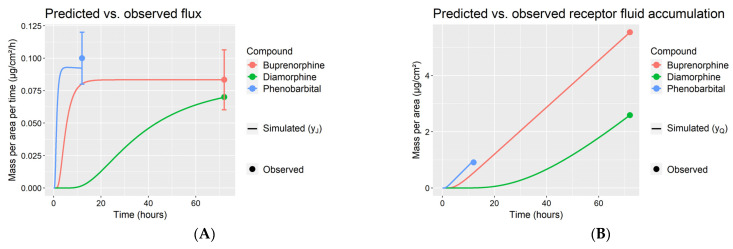
Observations and fitted dermal model simulations of flux (**A**) and receptor fluid accumulation (**B**). Error bars represent the mean observations ± one standard deviation.

**Figure 5:**
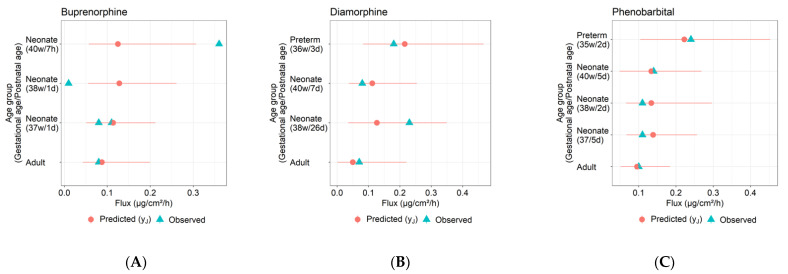
Observed and predicted (mean, 95% CI) flux for adults and newborns for (**A**) buprenorphine, (**B**) diamorphine, and (**C**) phenobarbital.

The model optimization and evaluation steps S1–S3 in Methods were implemented for each of buprenorphine, diamorphine, and phenobarbital. Figure 4 shows the step S2 fits of the adult dermal absorption model (where parameter Age=30 years) to the three compounds’ flux and receptor fluid measurements in Table 2. Figure 5 shows the predictive performance of the fitted model for each compound with respect to permeant flux across neonate and preterm infant skin, as generated by step S3. The pediatric dermal models described the general observed trends of higher dermal absorption (i.e., higher flux) in younger infants. For diamorphine and phenobarbital (Figure 5B,C), the dermal model provided reasonable predictions with most simulation outputs within the 95% confidence intervals. The fold error values of flux in neonates for diamorphine ranged from 0.55 to 1.4 (Table 7). For phenobarbital, the fold error values of flux rate in neonates ranged from 0.96 to 1.26. In terms of prediction for preterm neonates, the predicted flux was in good agreement with the observed data with fold error values of 1.2 and 0.93 for diamorphine and phenobarbital, respectively. For buprenorphine (Figure 5A), the model could not describe the inter-individual variability observed in full-term neonates (gestational ages of 38 and 40 weeks). The model predicted reasonably for an early term neonate (gestational age of 37 week) with 1–1.37-fold error.

### 3.4. Sensitivity Analysis

Post hoc sensitivity analysis was carried out to identify age-dependent parameters for which the outcomes were sensitive. The flux prediction showed age-dependent sensitivity to the SC thickness and a permeability-related parameter (i.e., $\log_{10}k_{trans})$, such that the absolute sensitivity coefficients of these parameters were higher in neonates than those seen in adults.

## 4. Discussion

In this study, the previously published dermal absorption model of Dancik et al. [2] was adapted to incorporate the maturation of skin anatomy and physiology in children. Through a literature review of physiological and anatomical skin parameters, it was found that that all skin layer thicknesses and the skin hydration state of the stratum corneum were age-dependent. Based on literature data, maturation equations were developed and incorporated into the model.

Adult-to-children PK extrapolation was performed using pediatric physiologically based pharmacokinetic modeling (e.g., [85,86]). In this workflow, adult models were first constructed by optimizing key chemical specific parameters using the observed PK data in adults. Then, the age-dependent components of the model such as hepatic clearance and protein binding are scaled for children. In light of the established workflow of pediatric physiologically based pharmacokinetic (PBPK) modeling, the same steps were followed in this study. The adult model was optimized using the observed data in adults (e.g., flux and cumulative amount) obtained from IVPT experimentation. While chemical-specific parameters in the model remained unchanged, age-dependent components of dermal absorption (e.g., skin layer thickness and SC hydration) were parameterized as a function of age in children.

The model adequately described the relative difference in dermal absorption between adults and infants that were observed in in vitro experiments. In general, infants tended to have a higher absorption rate with higher flux rates (J) compared to that of the adults for buprenorphine, diamorphine, and phenobarbital. In other words, the model successfully predicted the relative differences in dermal absorption between adults and children by taking into account the maturation of skin layer thicknesses and skin hydration.

The predicted flux values agreed with the observed values in neonates for diamorphine and phenobarbital. In the case of buprenorphine, a high inter-individual variability was observed in experimental results between the 40 week-7 h child and the 38 week-1 day child. The 30-fold difference in flux values between these two skin samples could not be captured by the model and this discrepancy is thought to be due to an experimental error. The improbable values could have been due to the handling of the skin samples before the experiment, such as the freezing, thawing and treating the samples with water for rehydration [29], causing the neonatal skin to become damaged.

Pre-term infants with a lower gestational age exhibited higher absorption rates compared to full-term neonates [29,30,31,87]. These trends were captured by the developed dermal absorption model accounting only for SC thickness being the difference between pre-term and term neonates. This speaks to the importance of SC thickness in driving flux rate in the model. The model could reasonably describe the flux rate in preterm neonates (i.e., GA: 35–36 weeks), with fold error values ranging from 0.93 to 1.2.

Clearly, these results provide only a small amount of evidence that the anatomy and physiology changes in the model are correct. This study as a whole is limited by the amount of in vitro skin penetration data available for this age group. However, the limited data that were found could generally be recapitulated by the model, although further experimentation would strengthen the basis to say that the model is predictive of age-related changes in dermal absorption.

The prediction of dermal absorption in children is critical for pediatric clinical applications. Transdermal drug delivery in neonates is advantageous because it can replace an invasive procedure of an intravenous line or an oral administration [88]. The smaller dose requirements and high permeability in neonates makes transdermal drug delivery more plausible [89]. This form of delivery has already been used in children—for example, fentanyl, tulobuterol and lindocaine:prilocaine (EMLA) [88].

Topical bioavailability can be determined by the physicochemical properties of a drug and the vehicle, such as: temperature, skin anatomy and physiology, skin hydration and metabolism in dermis and epidermis [90]. The stratum corneum plays an important role in dermal absorption as a skin barrier [21,88,91]. The lipid composition and integrity of the SC are important components in the regulation of skin permeability [5,92]. Therefore, the maturation of the SC determines the extent and the rate of dermal absorption in children [88]. The post hoc sensitivity analysis indicated that the flux rate prediction was the most sensitive to the SC thickness, indicating that the pediatric model appropriately reflected these literature findings. This was also corroborated in the flux prediction for preterm infants.

Compound-specific parameters of $\log_{10}k_{trans},\;K_{lip/w}\;and\;PC_{pro/w}$ were optimized based on the available dermal absorption data in adults (e.g., flux, cumulated amounts), and these parameters were kept the same in both adult and children models. According to the post hoc sensitivity analysis, the parameters that were the most important in predicting the relative difference of dermal absorption between adults and children was the SC thickness and $\log_{10}k_{trans}$. The satisfactory prediction accuracy of the model output indicated that the most important age-related parameters were appropriately parameterized in the model.

According to Code of Federal Regulations Title 21 Part 320 (§320.23) [93], it was stated that “For drug products that are not intended to be absorbed into the bloodstream, bioavailability may be assessed by measurements intended to reflect the rate and extent to which the active ingredient or moiety becomes available at the site of action”. In vitro permeation testing is an important tool for evaluating the permeation amount and the rate of active compounds with the use of excised human skin [94]. It is required to characterize the rate and extent of drug delivery via transdermal or topical routes to demonstrate bioequivalence [95]. The relative difference in dermal absorption between adults and children can be predicted by taking into account the physicochemical properties of the drug and the maturation of skin physiology and anatomy. With the available in vitro permeation test data in adults, this dermal model in children can provide an estimation of a rate of absorption (flux $y_{J}$) following topical exposure.

## Figures and Tables

**Table 1:** Compound-specific model input parameters.

Property	Buprenorphine	Diamorphine	Phenobarbital
Molecular formula	C29H41NO4	C21H23NO5	C12H12N2O3
Molecular weight (g/mol)	467.6	369.4	232.2
Lipophilicity (Log P)	4	1.5	1.47
Boiling point (°C)	578.7	272	
Melting point (°C)	217	173	174
Water Solubility (mg/L)	16.8	600	1110
Solubility in ethanol (mg/mL)	N/A	N/A	100
pKa	8.65 (basic)	7.83 (basic)	7.3 (acidic)
Vapor pressure (mmHg)	N/A	N/A	1.4 × 10^−11^

N/A: not available.

**Table 2:** Experimental conditions and observed permeant flux values in adults.

Compound (Reference)	Dose (µg/cm^2^)	Experiment Duration (h)	Solvent	Observed Steady-State Flux (µg/cm^2^/h)	Final Receptor Fluid Accumulation (µg/cm^2^)
Buprenorphine (Barret et al., 1994)	2656	72	0.1 M acetate buffer, pH 4	0.08 ± 0.02 (mean ± SD)	5.54
Diamorphine (Barret et al., 1993)	53,100	72	0.1 M acetate buffer, pH 4	0.07	2.59
Phenobarbital (Bonina et al., 1993)	78	12	Ethanol	0.1 ± 0.02	0.91

**Table 3:** Experimental conditions and observed permeant flux values in infants.

Compound (Reference)	Gestational Age (Postnatal Age)	Dose (µg/cm^2^)	Experiment Duration (h)	Solvent	Observed Steady-State Flux (µg/cm^2^/h)
Buprenorphine (Barret et al., 1994)	38 w (1 d)	2656	72	0.1 M acetate buffer pH 4	0.01
	40 w (7 h)				0.36
	37 w (1 d)				0.08
	37 w (1 d)				0.11
Diamorphine (Barret et al., 1993)	38 w (26 d)	53,100	72	0.1 M acetate buffer pH 4	0.23
	40 w (7 d)				0.08
	36 w (3 d)				0.18
Phenobarbital (Bonina et al., 1993)	38 w (2 d)	78	12	Ethanol	0.11
	40 w (5 d)				0.14
	37 w (5 d)				0.11
	35 w (2 d)				0.24

**Table 4:** Maturation ratio estimating equations.

Parameter	Equation	Coefficients
$\mathrm{Stratum}\;\mathrm{corneum}\;\mathrm{thickness}\;(P_{A_{1}}$)	*SC Maturation Model 1* $\frac{P_{A_{1}}\left(Age\right)}{P_{adult}}=a\cdot \;Age^{b}+c\cdot Age^{d}+e$ for Age ≤ 1510 days $\frac{P_{A_{1}}\left(Age\right)}{P_{adult}}=1$ for *Age* 1510 days	a= 2.401 × 10^−7^ b= 2.000 c= −99.43 d= 2.071 × 10^−3^ e= 101.4
	*SC Maturation Model 2* $\frac{P_{A_{1}}\left(Age\right)}{P_{adult}}=a\cdot \;Age^{b}+c$ for Age ≤ 1604 days $\frac{P_{A_{1}}\left(Age\right)}{P_{adult}}=1\;$for Age 1604 days	a= 2.662 × 10^−7^ b= 1.878 c= 0.724
$\mathrm{Epidermis}\;\mathrm{thickness}\;(P_{A_{2}}$)	$\frac{P_{A_{2}}\left(Age\right)}{P_{adult}}=\frac{\left(1-c\right)\cdot \;Age^{n}}{b^{n}+Age^{n}}+c$	b= 18.702 c= 0.634 n= 5.363
$\mathrm{Dermis}\;\mathrm{thickness}\;(P_{A_{3}}$)	$\frac{P_{A_{3}}\left(Age\right)}{P_{adult}}=\frac{\left(1.5-c\right)\;\cdot \;Age}{b+Age}+c$ for Age ≤ 9883 days $\frac{P_{A_{3}}(Age)}{P_{adult}}=1\;$for Age 9883 days	b= 8.974 × 10^3^ c= 0.407
$\mathrm{Stratum}\;\mathrm{corneum}\;\mathrm{hydration}\;(P_{A_{4}}$)	$\frac{P_{A_{4}}\left(Age\right)}{P_{adult}}=a\cdot \;Age^{n}+b\cdot Age^{m}+c$ for Age ≤ 1182 days $\frac{P_{A_{4}}\left(Age\right)}{P_{adult}}=1\;$for Age 1182 days	a= −0.344 b= −17.585 c= 18.530 n= 0.245 m= −0.0171

**Table 5:** Nominal values and uncertainties in stratum corneum parameters.

Parameter (Units)	Nominal Value (Uncertainty Range)	Source
$\log_{10}k_{trans}$(cm/s)	$\mathrm{Nominal}\;\mathrm{value}\;-0.570-0.840MW^{\frac{1}{3}}$ Uncertainty range = Nominal value ± 1.26	Wang et al., 2006 [76]
$\log_{10}PC_{pro/w}$	$\mathrm{Nominal}\;\mathrm{value}=0.27\log_{10}K_{o/w}+\log_{10}5.4$ Uncertainty range = Nominal value ± 0.32	Anderson and Raykar 1989 [77]
$\log_{10}K_{lip/w\;}$	$\mathrm{Nominal}\;\mathrm{value}=0.81\log_{10}K_{o/w}+\log_{10}0.43$ Uncertainty range = Nominal value ± 0.434	Nitsche et al., 2006 [78]

**Table 6:** Stratum corneum thickness measurements collected from the literature.

Mean (SD) SC Thickness (µm)	Skin Anatomical Site	Reference
13.2 (2.3)	Abdomen	Khiao In et al., 2019 [79]
21 (2.3)	Forearm	Choe et al., 2018 [80]
19 (1.3)	Forearm	
10.4 (3.2)	Forearm	Sauermann et al., 2002 [81]
11.2 (1.9)	Forearm	
13.3	Buttock	Therkildsen et al., 1998 [82]
18.3 (4.9)	Dorsal forearm	Sandby-Møller et al., 2003 [83]
11 (2.2)	Shoulder	
14.9 (3.4)	Buttock	
9.3	Back of hand	Robertson and Rees 2010 [84]
8.7	Centre of calf	
10.9	Outer forearm	
6.2	Inner forearm	
6.4	Inner upper arm	
8.4	Upper back	
6.5	Chest	
6.3	Abdomen	
5.8	Corner of eye	
6.3	Temple	

**Table 7:** Observed and predicted flux values in adults and infants.

Compound (Reference)	Gestational Age (Postnatal Age)	Observed Steady-State Flux (µg/cm^2^/h)	Predicted Terminal Flux (µm/cm^2^/h)		Predicted Geometric Mean/Observed Flux Ratio (Fold Error)
			Geometric Mean	95% CI	
Buprenorphine (Barret et al., 1994)	38 w (1 d)	0.01	0.13	0.06–0.26	12.8
	40 w (7 h)	0.36	0.12	0.06–0.31	0.35
	37 w (1 d)	0.08	0.11	0.05–0.21	1.37
	37 w (1 d)	0.11	0.11	0.05–0.21	1.0
Diamorphine (Barret et al., 1993)	38 w (26 d)	0.23	0.13	0.04–0.35	0.55
	40 w (7 d)	0.08	0.11	0.04–0.25	1.4
	36 w (3 d)	0.18	0.22	0.08–0.47	1.2
Phenobarbital (Bonina et al., 1993)	38 w (2 d)	0.11	0.13	0.07–0.3	1.22
	40 w (5 d)	0.14	0.13	0.05–0.27	0.96
	37 w (5 d)	0.11	0.14	0.07–0.26	1.26
	35 w (2 d)	0.24	0.22	0.1–0.45	0.93

h: hours, d: days, w: weeks.

## Data Availability

All data used in this study are available from published material cited in the manuscript.

## Supplementary Materials

The following are available online at https://www.mdpi.com/article/10.3390/pharmaceutics14010172/s1, Table S1. Stratum corneum (SC) thickness in infants and children, Table S2. Thickness of the viable epidermis in infants and children, Table S3. Thickness of the dermis in infants and children, Table S4. Stratum corneum (SC) hydration in infants and children, Table S5. Corneocyte volume fraction, Table S6. Follicle size and Volume, Table S7. Stratum corneum thickness literature review and search strategy, Table S8. Epidermis thickness literature review and search strategy, Table S9. Dermis thickness literature review and search strategy, Table S10. Stratum corneum hydration literature review and search strategy, Table S11. Corneocyte volume fraction literature review and search strategy, Table S12. Lipid and protein ratio literature review and search strategy, Table S13. Follicle size, density, volume literature review and search strategy, Table S14. Albumin concentration literature review and search strategy.
