# Supplementary material for: Development and Evaluation of an In Silico Dermal Absorption Model Relevant for Children
Source: Pharmaceutics. 2022 Jan 12;14(1):172. doi: 10.3390/pharmaceutics14010172 (PMC8780349; doi:10.3390/pharmaceutics14010172)
Supplement: Supplementary file 1 [file pharmaceutics-14-00172-s001.zip › pharmaceutics-1488528-supplementary.pdf]

pharmaceutics

MDPI

Supplementary Materials: Development and Evaluation of an In Silico Dermal Absorption Model Relevant for Children

Yejin Esther Yun, Daniella Calderon-Nieva, Abdullah Hamadeh and Andrea N. Edginton

Table S1. Stratum corneum (SC) thickness in infants and children.

Measurement Type	In vivo/ in vitro/ ex vivo & Room conditions	Body Area	Age	Notes	Trend	Reference
Hematoxylin and Eosin (H&E)	*Ex vivo*	Abdomen	Child (*n* = 30): average = 28 months/3.9 years (3 months – 11 years) Infant <3 months (*n* = 10): average = 17.1 days Adult (*n* = 10): average = 26 years (17-46 years)	Values don't represent an absolute value for SC thickness, values are just used for relative comparison	No significant differences in SC thickness between any of the groups Adult SC = 35.4 µm, SD = 6.4 Child 3mo – 11 years. = 28.8 µm, SD = 10.6 Infant SC = 35.4 µm, SD = 11.3	[1]
Confocal laser scanning microscopy	*In vivo* 22-28°C, 63-85% relative humidity	Buttock Upper thigh Ventral forearm	Full-term neonates (*n* = 15) Measurements (4 times per site) at 4-7 days, 1,3,6 months after birth Mothers of infants *n* = 15 (only measured ventral forearm)		Significant decrease in forearm skin observed from 4-7 days(~22µm) to 3 months of age (~10 µm). Similar changes in buttock and thigh skin but degree of decrease was relatively lower. SC thickness was almost the same as adults from 1 month of age in forearm skin. After 4-7 days SC thickness from all sites ranged from ~10-15 µm.	[2]
Modified starrett gauge, dry thickness of stratum corneum	*Ex vivo*	Hip	8 years – 65 years, *n* = 10 donors total (3 females, 7 males) one section from each donor	Focus was to obtain reproducible results from single donor not the accuracy of measurement. Cell layers were counted on at least 6 locations and averaged to determine purity of stratum corneum isolation.	SC thickness for the only 8 years old: 8.1 µm 30-45 years old SC thickness ranged from: 6.2 – 19.1 µm Sc thickness positively correlated with dry mass (r = 0.93). Can't correlate thickness with age given only one donor <30 years and one > 45 years	[3]
Reflectance Confocal Microscopy	*In vivo* No topical products and cosmetics for at least 24 h prior to study	44 adults (25-40 years): Caucasian Fitzpatrick skin type I–III (*n* = 25) and African American (*n* = 19) Infants (3-49 months old)	Upper inner arm (*n* = 142) Dorsal forearm (*n* = 151)	Children were grouped into 7 age bins (average *n* = 11 per age bin): All 44 adults (average age 32 years) were placed into one age bin	SC thickness increases until 4 years of age from ~8 to 14µm, where it becomes similar to adult SC thickness (~13-14 µm) SC thickness of dorsal forearm and inner arms are similar in thickness	[4]
Reflectance Confocal Microscopy	*In vivo*	Infants: 6-24 months Mothers: 25-46 years old N = 20 per group	Lower thigh	SC thickness was calculated from top of corneocyte layer to where granular cells can be detected	Infant SC thickness was on average 30% lower than that of adult: Infant: 7.3 ± 1.1 µm Adult: 10.5 ± 2.1 µm	[5]
Reflectance Confocal Microscopy	*In vivo* Avoid skin care products for 24 hours 20–25°C	Infants: 3-24 months (*n* = 52)	Upper inner arm Lower thigh	SC thickness is measured from top of corneocyte layer to granular cells	On average SC thickness was lower than adults. Upper inner arm: infant SC was ~ 18% thinner than adults (5.3±1.4µm vs 7.9±1.8µm)	[6]

	40-60% humidity	Adult mothers: 20-40 years (*n* = 27)<br><br>Asian Descent			Thigh: 34% thinner SC in infants<br>These thickness values are thinner than Caucasian counterparts found in [5]<br>No significant differences between male and female infants	
Optical Coherence Tomography	*In vivo*	Healthy female Japanese volunteers (*n* = 116)<br>Age: teens (14-17 years old) to sixties	Forehead<br>Cheek<br>Inner side of upper arm<br>Inner side of forearm<br>Outer side of forearm<br>Back of hand<br>Abdomen<br>Back<br>Inner side of femur<br>Inner side of leg<br>Shin	Note: Quantitative values were thinner than reported in conventional textbooks	Thickest SC: back of hand<br>Thinnest SC: shin<br>SC thickness showed no correlation to aging (unlike epidermal thickness).<br>No trend was observed in SC thickness as aging occurs.<br>Only cheek SC thickness became significantly less in the forties but in the sixties it returned to almost the same level as that in the twenties.<br>Back of hand also seems to increase with age, but not stat significant.	[7]
Histological sections, light microscopy	*Ex vivo*<br>Post-mortem skin samples	Infants (*n* = 169)<br>Gestation: 24-40 weeks<br>3 groups:<br>36-40 weeks (*n* = 88)<br>31-35 (*n* = 22)<br><31 (*n* = 59)	Upper abdominal margin of the midline	SC thickness was measured based on a scoring system:<br>Barely visible (0)<br>Thin layer (1)<br>Medium layer (2)<br>Thick layer (3)	SC thickness increases with gestational age, SC is very thin or barely there until 34 weeks gestational age.<br>After 34 weeks gestation, SC forms and most have medium to thick SC layer.<br>SC thickness in term babies doesn’t increase over 16 weeks.<br>More infants have SC in the thick or medium layer category after 6 weeks of age, but many also have the same scoring from 0-4 weeks of age. Very few full term babies have thin SC.<br>Histologically, the SC thickness also showed similar structure and thickness in term baby at 1 day old and 4 months (16 weeks) old	[8]

Table S2. Thickness of viable epidermis in infants and children.

Measurement Type	In vivo/ in vitro/ ex vivo & room conditions	Body Area	Age	Notes	Trend	Reference
Holtain Skin Caliper	*In vivo*	Dorsal forearm	Age: 8-89 years old (*n* = 145) No skin disorders		Measured whole skin thickness. Thickness: bell curve distribution with peak at 25 years old for women and 45 years old for men. Women's skin was always thinner than men skin after the peak of thickness for women at 25 years old.	[9]
Reflectance Confocal microscopy	*In vivo*	Lower thigh	Infants 6-24 months old (*n* = 20) Infant Mothers: 25-46 years old (*n* = 20)	Epidermal thickness calculated from the top corneocyte layer (thickness includes Stratum Corneum) to the top of the dermal papilla	Infant supra-papillary epidermis was on average 20% thinner than that of adults (29.7±3.4 μm versus 36.2±5.2 μm) Differences between child and adult were statistically significant	[5]
Histology, paraffin, hematoxylin and eosin	Skin biopsies *ex vivo*	Capillitium Forehead Cheeks Anterior neck Thorax Axilla Abdomen Back Gluteus Anterior arm Anterior forearm Palm Anterior leg, Anterior lower leg Sole	Divided into age groups: full term to 1 years 1-12 years 13-22 years 23-55 years 56-73 years 10 donors per age group, N = 750 skin biopsies	Open-source journal	No stat analysis shown. Appears total skin thickness increases with age. Epidermis thickens from 0-1 years old to adulthood (23-53 years old) in forehead, palm, and sole. For covered skin areas the epidermis thickness stays relatively constant from 0-1 years old. Epidermis thickness decreases from 1 to 12 years and reaches similar thickness to adults	[10]
Histology, paraffin, hematoxylin and eosin	Skin biopsies of healthy human skin at autopsy, *ex vivo*	Capitillium (epidermis and corium aka dermis)	Neonate: full term to 1 years Childhood: 1-12 years Puberty/adolescence: 13-22 years Adult: 23-55 years Elderly: 56-73 years N = 60 biopsy specimens, 12 samples per age groups	Open-source journal	Epidermal thickness was higher in infants less than 1years old (160.8 μm) then decreased in children aged 1-12 years old (98.3 μm). From then epidermal thickness increased into adulthood (158.7-174.6 μm), then decreased after 56 years old (112 μm). Percentage of each epidermal layer in whole epidermis also changes with age.	[11]
Reflectance confocal laser scanning microscopy	*In vivo* 20–25°C 40-60% humidity	Upper inner arm Lower thigh	Infants: 3-24 months (*n* = 52) Infant mothers: 20-40 years (*n* = 27)	Thickness of the suprapapillary epidermis (SPE) was measured from the top corneocyte layer to the top of the dermal papilla (epidermal thickness includes Stratum corneum thickness)	On average infant SPE thickness was lower than adults. Upper inner arm: infant SPE ~ 22% thinner Thigh: Infant SPE 8% thinner These thickness values are thinner than Caucasian counterparts found in [5].	[6]
Histology, light microscopy	*In vitro*	Human foreskin	Newborn: 2-5 days old Child: 3-11 years Old Adult: 17-58 years old	Two different donors for each age category: Newborn- 2 and 3 day old Child- 4 and 10 years old Adult- 15 and 26 years. old	After 1 week of submerged culture, epidermal thickness decreased with age. Adult keratinocytes formed 1-2 cell layer thick epidermis. Newborn epidermis was 6-8 cell layers thick Child epidermis was 4-5 cell layers thick. The same was seen when cultured at air-liquid interface.	[12]

Confocal laser scanning microscopy	*In vivo*<br>22-28°C<br>63-85% humidity	Full-term neonates (n = 15)<br>Measurements at 4-7 days, 1,3,6 months after birth<br>Infant Mothers: n = 15 (only ventral forearm measured)	Buttock<br>Upper thigh<br>Ventral forearm	Epidermal thickness determined in two ways:<br>1. Skin surface to top of dermal area (dermal papillae)<br>2. Skin surface to the bottom of epidermal layer (rete ridges)<br>Epidermis thickness includes SC thickness	Dermal papilla not observed up to 4-7 days post birth but gradually observed up to 3 months of age. Dermal papilla increases in size and number up to 6 months of age, but the most drastic changes occur in the first 3 months.<br>**Skin surface to top of dermal area:** Increase in epidermal thickness at each site from 4-7 days old to 1 month old. After 1 month, thickness was mostly the same as in adults (21 μm ±3):<br>Ventral forearm: ~20 μm to 25 μm<br>Buttock: ~21 μm to 26 μm<br>Thigh: similar thickness from 4-7days to 1 month<br>**Epidermal thickness from skin surface to bottom of the epidermal layer, i**ncreased significantly between 4 to 7 days and 1 month of age in all areas. This change with growth after birth almost stopped before 1 month of age, and from 1 month of age, epidermal thickness from the skin surface to the bottom of the epidermal layer was mostly the same as in adults (ventral forearm only: ~60μm).<br>Ventral forearm: ~25μm at 4-7 days to 55μm at 1 month old	[2]
Histological sections, light microscopy	*Ex vivo*	Upper abdominal margin of the midline	Gestation at birth ranged from 24-40 weeks<br>3 groups:<br>36-40 weeks (n = 88)<br>31-35 (n = 22)<br><31 (n = 59)		Epidermal thickness increases with increasing gestational age<br>Epidermis increases with increasing postnatal age. The increase in epidermal thickness stops after 16 wks. of age<br>The undulation of the epidermis also increases with increased age post birth.<br>Premature babies develop epidermis in 2 weeks of life with increase thickness and cell layers. However, the undulation of the epidermis does not develop during this time.	[8]
Histology sections	Biopsy specimens	Parietal scalp	Age: 2 weeks – 21 years (n = 100)<br>62 males, 38 females	Minimum and maximum epidermis thickness was measured	Minimum epidermis thickness did not appear to vary with age (25μm).<br>But maximal epidermal thickness increased slightly with age (80 μm at birth to 160 μm at 21 years old):<br>Linear regression: 80.5 + 3.0y<br>R = 0.5, p<0.0001<br>Units in μm	[13]

Table S3. Thickness of Dermis in infants and children.

Method of measurement	In vivo/ in vitro/ ex vivo & room conditions	Body Area	Age	Notes	Trend	Reference
Histology sections	Biopsy specimens	Parietal scalp	Post-natal Age: 2 weeks – 21 years (*n* = 100) 62 males, 38 females	Dermis measured from dermal papilla to least and most prominent projection of collagen fibres. Adventitial dermis not considered	Both the minimum and maximum dermis thickness values increased with age (y = years) in parallel (units = μm) Minimum Dermis: <1-year-old: 850 μm 21 years old: 1500 μm Maximum Dermis: <1-year-old: 1125 μm 21 years old: 2200 μm **Regression Lines:** Min dermis: 777.5 + 32.9y (r = 0.64, p<0.0001) Max dermis: 1143.1 + 34.2y (r = 0.53, p<0.0001)	[13]
Histology, paraffin, hematoxylin and eosin	Skin biopsies	Capillitium Forehead Cheeks Anterior neck Thorax Axilla Abdomen Back Gluteus Anterior arm Anterior forearm Palm Anterior leg Anterior lower leg Sole	Divided into age groups: full term to 1 year 1-12 years 13-22 years 23-55 years 56-73 years N = 10 per age group	Open-source journal No in-text references	No stat analysis shown. Appears total skin thickness increases with age. Dermis thickness is higher in the 1-12 age group compared to the 0-1 year old age group at all regions measured except capillitium (scalp). Dermis also is higher in the 13-22 age groups compared to the 1-12 age group After this age, unsure whether it stays the same given no statistical analysis.	[10]
20 MHz sonography (allows depiction of the dermis, but not separate resolution of the epidermis)	*In vivo*	Cheek Volar forearm Thigh Calf	N = 310 7 age groups (*n* = 21-24 per group): 1wk ±5 days old 4weeks± 7 days old 6 months ± 20 days old 9months ± 20 days old 12 months ± 20 days old 24 months ± 84 days old 36months ± 84 days old	Did not mention the bounds of the dermis that were measured to get thickness values.	Measured **total skin thickness.** Dermal thickness at cheek appears highest at 1 week of age (1200μm) then slightly decreases at 4 weeks of age (1100μm), where it is similar from 6- 36 months of age (1000μm). Dermal thickness at forearm appears highest at 1 week of age (1200μm) then slightly decreases at 4 weeks of age (1100μm), where it is similar from 6- 36 months of age (1050μm)	[14]
Histology and microscopy	*Ex vivo*	Abdominal skin: periumbilical region adjacent to the midline. Area assumed to be protected from sun exposure	N = 45 Age 5 months old to 95 years Old Average age: 57.02 ± 27.68 years		**Thickness of papillary dermis increases with age.** It followed a linear regression model in a statistically significant way ($P < 0.0001$, $R^2 = 0.26$): from 63.24-μm-thick in the moment of birth until 100 μm at 100 years old (predicted). Reticular dermis thickness followed a quadratic function: $P = 0.011$, $R^2 = 0.193$	[15]

				The thickness was minimum in the first and last stages of life, with values of 1603.88 μm at the moment of birth and 1303.48 μm predicted for 100-year-olds, and maximum values in adult skin, reaching a thickness of 3236.18 μm at 50 years of age.	

Table S4. Stratum corneum (SC) hydration in infants and children.

**Method of measurement**	**In vivo/ in vitro/ ex vivo & room conditions**	**Body Area**	**Age**	**Trend**	**Reference**
Corneometer	24°C<br>50% humidity<br>*In vivo*	Cheek<br>Forearm	10-14 years old (N = 32)<br>Mothers: 40±4 years old	Skin hydration is lower in children compared to adults.<br>Cheek Summer (AU):<br>Children: 45.7±8.4<br>Adult: 56.3±8.8 P<0.0001<br>Cheek Winter (AU):<br>Children: 31.8±11.2<br>Adult: 49.2±9.9 P<0.0001<br>Forearm Summer<br>Children: 30.7±3.3<br>Adult: 37.2±5.0 P<0.0001<br>Forearm Winter<br>Children: 27.7±3.2<br>Adult: 31.2±5.0 P = 0.0026	[16]
Capacitance	20.6±0.62°C35.71±6.51 % humidity<br>*In vivo*	Forearm<br>Thigh	Birth (<72h old)- 4 weeks old (N = 39)<br>Compared to unrelated adults (N = 20)	Skin hydration increases significantly from birth<br>Birth: 17·66 ± 4·55 relative capacitance units (RCU)<br>Early infancy (4 weeks): 41·79 ± 9·65 RCU<br>Newborn infant skin was dryer than adults<br>17.66 RCU (infants) vs 31.47± 6.9 RCU (adults)	[17]
Capacitance	*In vivo*	Forearm	6.4 ± 0.31 years old<br>Eczema versus no eczema	Skin hydration measured as 62.29 ± 6.34 AU	[18]
Corneometer	20 ± 2 °C<br>50 ± 10% humidity<br>*In vivo*	Forearm	Age groups (*n* = 18 per group):<br>1-15 days old<br>5-6 weeks old<br>6± 1 months Old<br>1-2 years old<br>4-5 years old<br>Adult: 20-35 years	Mean skin hydration value for newborns 1-15 days old (17.4AU) was significantly lower compared with older age groups.<br>The 5-6 week old (41.2AU) and the 6 month old age group (41.5AU) had higher skin hydration compared to the other age groups.	[19]
Corneometer	21-25°C<br>Mean Humidity = 43.6%<br>Range humidity = 33-55% humidity<br>*In vivo*	Forearm	1-6 years old (*n* = 44)<br>Compared to adult parent 21-44 years old (*n* = 44)	Skin hydration not significantly different between children and adults.<br>Child: 75.4 ± 11.4 AU<br>Adult: 76.1 ± 8.4 AU	[20]
Corneometer	22-26°C<br>40-60% humidity<br>*In vivo*	Upper arm<br>Thigh<br>Buttock	6-24 months of age (*n* = 60)	No significant differences in skin hydration at any site from 6 months to 24 months of age.	[21]
Corneometer	*In vivo*	Forehead<br>Abdomen<br>Upper leg<br>Buttock	Newborns ≤48h old followed to 8 weeks of age (*n* = 64)	Hydration increased with age at all four sites.<br>Hydration in 2-day old neonate was ~25 AU and increased to ~55AU at 8 weeks of age.	[22]
Corneometer	21.6 ±1 .5°C<br>46.4 ± 7.45% humidity<br>*In vivo*	Forehead<br>Cheek<br>Forearm<br>Gluteal surface	3 days old newborns (*n* = 202)<br>Followed up at 4 and 12 weeks<br>Mothers: 18-40 years old	Hydration increased significantly at all tested sites during the neonatal period then remained stable after 30 days post birth.	[23]
Corneometer	*In vivo*	Forehead<br>Abdomen	Neonates ≤48h old followed until 4 weeks old.	Hydration significantly increased from day 2 -7 post birth.	[24]

		Upper leg<br>Buttock	Product applied after 7 days	The highest increase in hydration was in the abdomen, which increased by 7 AU.<br>An overall increase in hydration is also seen from age 2 days to 28 days old in the control group bathed with water and the group bathed with a washcloth and water.	
Corneometer	22–24°C<br>45–55% Humidity<br>*In vivo*	Forehead<br>Forearm	0.5 - 94 years of age<br>N = 713	Skin hydration increases to age 40-50 years then decreases	[25]
Corneometer	25°C<br>40% Humidity<br>*In vivo*	Upper thigh<br>Diaper covered buttock	Neonates 3 days old followed up to 1 year old (*n* = 19)<br>Mothers (*n* = 5)	Skin hydration is lower than adults in the thigh and buttock, then increases rapidly until 1 month of age and remains higher than adult hydration throughout the first year of life.	[26]
High frequency conductance	22-26°C<br>40-53% Humidity<br>*In vivo*	Forearm	Newborn infants 0-5 days old (*n* = 46)<br>Children 1-5 years old (*n* = 16)<br>Adults 22-47 years old (*n* = 10)	Newborns had lower conductance than adults. Conductance was higher in 1 month and 2-month-old infants compared to newborns.	[27]
Dermal Phase Meter	*In vivo*	Non-diapered skin above waistband<br><br>Forearm (mother)	Newborn infants (*n* = 31)<br>Followed from one day old to 28 days of age<br><br>Mothers	Skin surface hydration of non-diapered region increases in the first 2 postnatal weeks then plateaus.<br>Adult value (4.76 Log capacitive reactance) was higher than value in the first week of life but lower than value at one month of age.	[28]
Corneometer	22°C to 24°C<br>50% humidity<br>*In vivo*	Forehead<br>Upper black<br>Forearm<br>Palm<br>Abdomen<br>Inguinal region<br>Soles	Newborns (*n* = 44)<br>5-10 hours post-partum and followed until 24 hours post-partum<br><br>Adults: mean age of 24 (*n* = 20)	Hydration was significantly lower in neonates compared with adults in forehead, back, abdomen.<br>Hydration was significantly higher in neonates than adults in forearm and palms.	[29]
Corneometer	24–26°C<br>40–60% humidity<br>*In vivo*	Inner upper arm<br>Buttock<br>Cheek	Infants 2-24 months old (*n* = 63)<br>Mothers (*n* = 60)	Water content decreased in an age-dependent manner:<br>Infants 2-12 months old had highest capacitance<br>13–24-month-old group:<br>**Buttock:** higher water content than mothers<br>**Upper arms and cheeks:** significantly lower water content than mothers	[30]
Corneometer	16–20°C<br>44–47% humidity<br>*In vivo*	Dorsal Hand<br>Forehead<br>Canthus	0.15 -79-year-old volunteers (*n* = 633)<br>125 volunteers aged 0-10 years old	Increased hydration from the first decade of life to ~40 years, then decreases.	[31]

AU: arbitrary units; RCU: relative capacitance units.

Table S5. Corneocyte Volume Fraction.

Method of measurement	In vivo/ in vitro/ ex vivo & room conditions	Body Area	Age	Notes	Trend	Reference
Scanning Electron Microscopy	Adhesive disc to isolate and analyze surface of Stratum Corneum<br>20 ± 2 °C<br>50 ± 10% relative humidity	Volar forearm	1 day old to 5 years old<br>Adult patients were parents of children whenever possible.<br>6 age groups created (n = 6 or 5 per group):<br>(i) full term newborns (1–15 days; mean 0·3 months),<br>(ii) young babies (5–6 weeks old; mean 1·5 months),<br>(iii) older babies (6 ± 1 months old; mean 6·2 months),<br>(iv) young children (2 years old; mean 22·7 months),<br>(v) older children (4–5 years old; mean 50·4 months),<br>(vi) adults (20–35 years old; mean 336·0 months)	Not a direct measure of corneocyte volume fraction of SC.<br>1-2 year old age group was analysed separately given larger age gap in enrolled individuals<br>Developed isotropy score based on SEM images.<br>Score parameters: (i) cell density (× 30 magnification); (ii) cluster formation (× 30 magnification); (iii) cell shape and adhesion (× 500 magnification); and (iv) resolution (× 500 magnification)<br>Score from 0-3 points per parameter (12 max per sample), total score divided into 3 categories: anisotropy(immature), intermediate isotropy, good isotropy (mature)	Correlation: Younger age group had lower score. Child 6 months to 4-5 years had lower scores than adults too.<br>Under age 2, change in score was very fast, then the rate increased at a slower rate to adult hood.<br>Irregular corneocyte distribution was observed under age 1, and the projected area showed a progressive age dependent increase.<br>Overall: Skin matured quickly until age 2 years old, then slows until adulthood in the morphology of the corneocytes.	[32]
Atomic Force Microscopy	*In vitro*<br>Tape striped corneocyte from surface of skin<br>Indoor ordinary environment	Cheek<br>Flexor surface of upper arm	N = 12 females<br>Age: 1 -82 years old<br><br>7 participants were < 20 years old:<br>1 years old: n = 1<br>2 years old: n = 2<br>4 years old: n = 1<br>9 years old: n = 2<br>16 years old: n = 1	2D and 3D parameters of corneocytes were measured:<br>Average thickness<br>Projected cell surface area (as it increases, cell turnover decreases)<br>Real surface area<br>Volume<br>Flatness Index (projected area/average thickness X 10^{-3})	Average corneocyte thickness was greater in the upper arm than the cheek regardless of age.<br>Corneocyte thickness decreased with age for the upper arm but not for the cheek due to too much individual variation.<br>Projected surface area of corneocytes in the flexor upper arm increased with age (relationship was not as prominent in the cheek). Corneocytes from forearm were larger than the cheek.<br>Flatness index of forearm corneocytes also increased with age (as age increases, corneocytes become bigger and flatter). This was not observed for cheek corneocytes.	[33]
Reflectance Confocal Microscopy	*In vivo*<br>One tape strip of surface corneocytes	Infants 6-24 months<br>Mothers 25-46 years old<br>N = 20 per group	Upper inner arm<br>Dorsal forearm<br>Lower thigh area		Size of infant corneocytes was smaller than adult corneocytes at all sites.<br>The size of corneocytes between the different sites were not significantly different in adults or children.<br>Corneocyte size (μm²):<br>Upper inner arm:<br>Infant: 949.9 ± 19.1<br>Adult: 1077.6 ± 26.9<br>Dorsal forearm:<br>Infant: 907.3 ± 23.4<br>Adult: 1071.0 ± 25.7	[5]

					Thigh:<br>Infant: 953.0 ± 23.8<br>Adult: 1154.4 ± 33.7<br>Smaller size of corneocytes was attributed to higher cell proliferation rate.<br>Volume of corneocytes from the upper arm were higher than the cheek corneocytes.<br>No clear correlation between the volume of corneocytes and the age of the participants in cheek or upper arm samples due to large individual variations at both sites.	
Several	*In vivo*	8 years old – 89 years old	Ventral side of arm	The main focus is older age.<br>Measured number of corneocytes and surface area	Corneocyte count increases linearly with age. Number of corneocytes increase very sharply after 60 years of age.<br>Projected surface area of corneocytes increases with age.	[9]

Table S6. Follicle Size and Volume.

**Method**	**In vivo/ in vitro/ ex vivo & room conditions**	**Body Area**	**Age**	**Notes**	**Trend**	**Ref.**
Transmission Electron Microscopy<br>Scanning Electron Microscopy	3mm Punch biopsies for only 6 infants without Erythema toxicum	Lower leg	Infants:<br>1 day old (≥24h <48h) (n = 69)<br>Adults: n = 4	None of the babies were bathed before sample collection	The number of visible hair structures/mm^2 was 3.5 ± 0.08 in infants (n = 2) and 0.3 ± 0.15 (mean ± SD) in adults (n = 4)<br>Newborn infants have ~10 times more hair follicles than adults (comes from a textbook stating that newborns have approximately 5 million hair follicles on their body but no reference to this number [34])	[35]
Histology, hematoxylin and eosin	*Ex vivo*		Neonatal full-term infants to elderly up to 73 years old	Unpublished data	Length and width of hair follicles changes with age<br>Follicle diameter increases from 0.13mm to 0.558 in elderly. In newborns the length of longitudinal follicle is 1.113 and increases up to 4.5mm in adolescence	[11]

Table S7. Stratum Corneum Thickness Literature Review and Search Strategy.

The following search strategy was inputted into PubMed to identify publications that contained quantitative stratum corneum thickness measurements in infants and children.

**Input**	**Results**
"stratum corneum" AND (thick* OR thickness OR depth OR deep OR width OR thin*) AND (development OR develop* OR time OR life OR growth OR progress OR change OR age OR maturation)	1191
English language and full text filters	1039
Exclude review articles	980
Human only filter	609
Relevant articles	43
Final Selected	17

Table S8. Epidermis Thickness Literature Review and Search Strategy.

The following search strategy was inputted into PubMed to identify publications that contained quantitative epidermis thickness measurements in infants and children.

Input	Filters	Results
(Epidermis [mesh] OR "Epidermal cells" OR epidermal) AND (Thick* OR thickness OR depth OR deep OR width OR thin*) AND (Development [tiab] OR develop*[tiab] OR time OR life OR growth OR progress OR change [tiab] OR maturation [tiab] OR matur*) NOT "stem cell" NOT "growth factor" NOT burn NOT graft NOT Langerhans NOT review [pt] NOT treatment [tiab]	English Human Full-Text	1262
Relevant articles		27
Final Selected		9

Table S9. Dermis Thickness Literature Review and Search Strategy.

The following search strategy was inputted into PubMed to identify publications that contained quantitative dermis thickness measurements in infants and children.

Input	Filters	Results
(Dermis [mesh] OR papillary dermis) AND (Thick* OR thickness OR width OR depth[tiab] OR thin*) AND (Development [tiab] OR develop*[tiab] OR time [tiab] OR "early life" OR growth [tiab] OR progress* OR change [tiab] OR age OR maturation [tiab])	English Human Full-Text	509
Relevant articles		7
Final Selected		4

Table S10. Stratum Corneum Hydration Literature Review and Search Strategy.

The following search strategy was used to search MEDLINE and EMBASE using OVID to identify publications that quantitatively measured the water content or fraction in the stratum corneum of infants or children.

Number	Searches	EMBASE Results	MEDLINE Results
1	horny layer.mp. [mp=title, abstract, heading word, drug trade name, original title, device manufacturer, drug manufacturer, device trade name, keyword, floating subheading word, candidate term word]	838	697
2	cornified cell envelope.mp. [mp=title, abstract, heading word, drug trade name, original title, device manufacturer, drug manufacturer, device trade name, keyword, floating subheading word, candidate term word]	267	219
3	exp stratum corneum/	11603	0
4	stratum corneum.mp. [mp=title, abstract, heading word, drug trade name, original title, device manufacturer, drug manufacturer, device trade name, keyword, floating subheading word, candidate term word]	15894	9335
5	hydration.mp. or exp hydration/ or exp skin hydration meter/	54620	36122
6	chemical composition/ or exp lipid composition/ or exp tissue water/ or exp water content/	123638	0
7	(water adj4 (content or fraction or percent*)).mp. [mp=title, abstract, heading word, drug trade name, original title, device manufacturer, drug manufacturer, device trade name, keyword, floating subheading word, candidate term word]	46162	33771
8	exp infant/	985694	1139847
9	(infant* or newborn* or full term or neonat* or child or children).mp. [mp=title, abstract, heading word, drug trade	3279134	3096201

	name, original title, device manufacturer, drug manufacturer, device trade name, keyword, floating subheading word, candidate term word]		
10	1 or 2 or 3 or 4	16569	10068
11	5 or 6 or 7	203833	68485
12	8 or 9	3283560	3096201
13	10 and 11 and 12	219	134
14	limit 13 to (human and english language)	171	101
	Duplicates	82	
	Final Selection	15	

Table S11. Corneocyte Volume Fraction Literature Review and Search Strategy.

The following search strategy was used to search MEDLINE and EMBASE using OVID to identify publications that quantitatively measured size, volume, or surface area of corneocytes in the stratum corneum of infants or children.

Number	Searches	EMBASE Results	MEDLINE Results
1	horny layer.mp. [mp=title, abstract, heading word, drug trade name, original title, device manufacturer, drug manufacturer, device trade name, keyword, floating subheading word, candidate term word]	838	697
2	cornified cell envelope.mp. [mp=title, abstract, heading word, drug trade name, original title, device manufacturer, drug manufacturer, device trade name, keyword, floating subheading word, candidate term word]	267	219
3	exp stratum corneum/	11603	0
4	stratum corneum.mp. [mp=title, abstract, heading word, drug trade name, original title, device manufacturer, drug manufacturer, device trade name, keyword, floating subheading word, candidate term word]	15892	9334
5	corneocyte.mp. [mp=title, abstract, heading word, drug trade name, original title, device manufacturer, drug manufacturer, device trade name, keyword, floating subheading word, candidate term word]	639	416
6	(corneocyte adj3 size).mp. [mp=title, abstract, heading word, drug trade name, original title, device manufacturer, drug manufacturer, device trade name, keyword, floating subheading word, candidate term word]	36	25
7	(corneocyte adj6 volume).mp. [mp=title, abstract, heading word, drug trade name, original title, device manufacturer, drug manufacturer, device trade name, keyword, floating subheading word, candidate term word]	4	1
8	(corneocyte adj6 phase).mp. [mp=title, abstract, heading word, drug trade name, original title, device manufacturer, drug manufacturer, device trade name, keyword, floating subheading word, candidate term word]	11	9
9	(corneocyte adj6 fraction).mp. [mp=title, abstract, heading word, drug trade name, original title, device manufacturer, drug manufacturer, device trade name, keyword, floating subheading word, candidate term word]	1	1
10	*infant/ or *Infant, Newborn/	43411	18885
11	(full term or neonat* or child).mp. [mp=title, abstract, heading word, drug trade name, original title, device manufacturer, drug manufacturer, device trade name, keyword, floating subheading word, candidate term word]	2580392	2371036
12	1 or 2 or 3 or 4	16567	10067
13	5 or 6 or 7 or 8 or 9	639	416
14	10 or 11	2599884	2379691
15	12 and 13 and 14	21	20
	Duplicates	14	
	Total Selected	26	
	Final Selection	4	

Table S12. Lipid and Protein Ratio Literature Review and Search Strategy.

The following search strategy was used to search EMBASE and MEDLINE using OVID to identify publications that quantitatively measured the lipid and protein quantities in the stratum corneum of infants or children.

Number	Search	Results
1	exp lipid/ or exp lipid bilayer/ or exp skin lipid/	1548059
2	(content* or fraction or amount).mp. [mp=title, abstract, heading word, drug trade name, original title, device manufacturer, drug manufacturer, device trade name, keyword, floating subheading word, candidate term word]	1980619
3	*stratum corneum/	2843
4	(horny layer or cornified envelope).mp. [mp=title, abstract, heading word, drug trade name, original title, device manufacturer, drug manufacturer, device trade name, keyword, floating subheading word, candidate term word]	1661
5	(infant* or neonat* or child*).mp. [mp=title, abstract, heading word, drug trade name, original title, device manufacturer, drug manufacturer, device trade name, keyword, floating subheading word, candidate term word]	3213267
6	exp pediatrics/	106644
7	3 or 4	4338
8	5 or 6	3237629
9	exp phospholipid/	191370
10	free fatty acid.mp. or fatty acid/	113305
11	lipid matrix.mp. [mp=title, abstract, heading word, drug trade name, original title, device manufacturer, drug manufacturer, device trade name, keyword, floating subheading word, candidate term word]	1305
12	1 or 9 or 10 or 11	1550458
13	2 and 7 and 8 and 12	22
Final Selection	2	

*Medline: no results for final search.

Table S13. Follicle Size, Density, Volume Literature Review and Search Strategy.

The following search strategy was used to search MEDLINE and EMBASE using OVID to identify publications that quantitatively measured follicle physical properties in the skin of infants or children.

Number	Searches	EMBASE Results	MEDLINE Results
1	exp Infant/	985694	1138991
2	(neonat* or newborn* or child or children or infant* or preschool age or school age).mp.	3274956	3089674
3	exp Hair Follicle/	14897	6400
4	(hair? adj3 (follicle? or appendage? or structure?)).mp.	20705	14465
5	(count or counted or number or microscopy).mp.	3880791	2945093
6	exp Microscopy/	824006	549293
7	1 or 2	3279469	3089674
8	3 or 4	20705	18177
9	5 or 6	3893008	2955727
10	7 and 8 and 9	496	327
11	limit 10 to (english language and humans)	313	190
	Duplicates	117	
	Final selection	2	

Table S14. Albumin Concentration Literature Review and Search Strategy.

The following search strategy was used to search MEDLINE and EMBASE using OVID to identify publications that quantitatively measured albumin content or fraction in the epidermis of infants or children.

Number	Searches	EMBASE Results	MEDLINE Results
1	exp albumin/ or exp albumin level/	131881	0
2	exp epidermis/	159372	27270
3	exp infant/	946163	1151595
4	newborn*.mp. [mp=title, abstract, heading word, drug trade name, original title, device manufacturer, drug manufacturer, device trade name, keyword, floating subheading word, candidate term word]	614911	768034
5	(full term or neonat* or child or children).mp. [mp=title, abstract, heading word, drug trade name, original title, device manufacturer, drug manufacturer, device trade name, keyword, floating subheading word, candidate term word]	2728408	2613394
6	3 or 4 or 5	3089382	3050030
7	1 and 2 and 6	39	0
Final Selection	1		

References

1. Fairley JA, Rasmussen JE: Comparison of stratum corneum thickness in children and adults. *Journal of the American Academy of Dermatology* 1983, 8:652-654.
2. Miyauchi Y, Shimaoka Y, Fujimura T, Koike Y, Yatabe M, Nishikawa M, Hayashi M, Sugata K, Moriwaki S, Hatamochi A: Developmental Changes in Neonatal and Infant Skin Structures During the First 6 Months: In Vivo Observation. *Pediatr Dermatol* 2016, 33:289-295.
3. Anderson RL, Cassidy JM: Variation in physical dimensions and chemical composition of human stratum corneum. *J Invest Dermatol* 1973, 61:30-32.
4. Walters RM, Khanna P, Chu M, Mack MC: Developmental Changes in Skin Barrier and Structure during the First 5 Years of Life. *Skin Pharmacology and Physiology* 2016, 29:111-118.
5. Stamatas GN, Nikolovski J, Luedtke MA, Kollias N, Wiegand BC: Infant Skin Microstructure Assessed In Vivo Differs from Adult Skin in Organization and at the Cellular Level. *Pediatric Dermatology* 2010, 27:125-131.
6. Liu Q, Zhang Y, Danby SG, Cork MJ, Stamatas GN: Infant Skin Barrier, Structure, and Enzymatic Activity Differ from Those of Adult in an East Asian Cohort. *Biomed Res Int* 2018, 2018:1302465.
7. Tsugita T, Nishijima T, Kitahara T, Takema Y: Positional differences and aging changes in Japanese woman epidermal thickness and corneous thickness determined by OCT (optical coherence tomography). *Skin Res Technol* 2013, 19:242-250.
8. Evans NJ, Rutter N: Development of the epidermis in the newborn. *Biol Neonate* 1986, 49:74-80.
9. Leveque JL, Corcuff P, de Rigal J, Agache P: In vivo studies of the evolution of physical properties of the human skin with age. *Int J Dermatol* 1984, 23:322-329.
10. Kakasheva-Mazhenkovska L, Milenkova L, Gjokik G, Janevska V: Variations of the histomorphological characteristics of human skin of different body regions in subjects of different age. *Prilozi* 2011, 32:119-128.
11. Kakasheva-Mazhenkovska L, Milenkova L, Kostovska N, Gjokik G: Histomorphometrical characteristics of human skin from capillitium in subjects of different age. *Prilozi* 2011, 32:105-118.
12. sMichel M, L'Heureux N, Auger FA, Germain L: From newborn to adult: phenotypic and functional properties of skin equivalent and human skin as a function of donor age. *J Cell Physiol* 1997, 171:179-189.
13. de Viragh PA, Meuli M: Human scalp hair follicle development from birth to adulthood: Statistical study with special regard to putative stem cells in the bulge and proliferating cells in the matrix. *Archives of Dermatological Research* 1995, 287:279-284.
14. Hughes-Formella B, Wunderlich O, Williams R, Fernández J, Kim YZ, Wigger-Alberti W, Pecquet S, Moodycliffe A: Comparison of Skin Structural and Functional Parameters in Well-Nourished and Moderately Undernourished Infants. *Skin Pharmacology and Physiology* 2019, 32:212-223.
15. Marcos-Garcés V, Molina Aguilar P, Bea Serrano C, García Bustos V, Benavent Seguí J, Ferrández Izquierdo A, Ruiz-Saurí A: Age-related dermal collagen changes during development, maturation and ageing - a morphometric and comparative study. *J Anat* 2014, 225:98-108.

16. Akutsu N, Ooguri M, Onodera T, Kobayashi Y, Katsuyama M, Kunizawa N, Hirao T, Hosoi J, Masuda Y, Yoshida S, et al: Functional characteristics of the skin surface of children approaching puberty: age and seasonal influences. *Acta Derm Venereol* 2009, 89:21-27.
17. Chittock J, Cooke A, Lavender T, Brown K, Wigley A, Victor S, Cork MJ, Danby SG: Development of stratum corneum chymotrypsin-like protease activity and natural moisturizing factors from birth to 4 weeks of age compared with adults. *Br J Dermatol* 2016, 175:713-720.
18. Eberlein-König B, Schäfer T, Huss-Marp J, Darsow U, Möhrenschlager M, Herbert O, Abeck D, Krämer U, Behrendt H, Ring J: Skin surface pH, stratum corneum hydration, trans-epidermal water loss and skin roughness related to atopic eczema and skin dryness in a population of primary school children. *Acta Derm Venereol* 2000, 80:188-191.
19. Fluhr JW, Darlenski R, Lachmann N, Baudouin C, Msika P, De Belilovsky C, Hachem JP: Infant epidermal skin physiology: adaptation after birth. *Br J Dermatol* 2012, 166:483-490.
20. Fluhr JW, Pfisterer S, Gloor M: Direct Comparison of Skin Physiology in Children and Adults with Bioengineering Methods. *Pediatric Dermatology* 2000, 17:436-439.
21. Fujimura T, Miyauchi Y, Shima K, Hotta M, Tsujimura H, Kitahara T, Takema Y, Palungwachira P, Laohathai D, Chanthothai J, Nararatwanchai T: Ethnic differences in stratum corneum functions between Chinese and Thai infants residing in Bangkok, Thailand. *Pediatric Dermatology* 2018, 35:87-91.
22. Garcia Bartels N, Scheufele R, Prosch F, Schink T, Proquitté H, Wauer RR, Blume-Peytavi U: Effect of Standardized Skin Care Regimens on Neonatal Skin Barrier Function in Different Body Areas. *Pediatric Dermatology* 2010, 27:1-8.
23. Hoeger PH, Enzmann CC: Skin Physiology of the Neonate and Young Infant: A Prospective Study of Functional Skin Parameters During Early Infancy. *Pediatric Dermatology* 2002, 19:256-262.
24. Garcia Bartels N, Mleczko A, Schink T, Proquitté H, Wauer RR, Blume-Peytavi U: Influence of bathing or washing on skin barrier function in newborns during the first four weeks of life. *Skin Pharmacol Physiol* 2009, 22:248-257.
25. Man MQ, Xin SJ, Song SP, Cho SY, Zhang XJ, Tu CX, Feingold KR, Elias PM: Variation of skin surface pH, sebum content and stratum corneum hydration with age and gender in a large Chinese population. *Skin Pharmacol Physiol* 2009, 22:190-199.
26. Minami-Hori M, Honma M, Fujii M, Nomura W, Kanno K, Hayashi T, Nakamura E, Nagaya K, Miyauchi Y, Fujimura T, et al: Developmental alterations of physical properties and components of neonatal-infantile stratum corneum of upper thighs and diaper-covered buttocks during the 1st year of life. *Journal of Dermatological Science* 2014, 73:67-73.
27. Saijo S, Tagami H: Dry skin of newborn infants: functional analysis of the stratum corneum. *Pediatr Dermatol* 1991, 8:155-159.
28. Visscher MO, Chatterjee R, Munson KA, Pickens WL, Hoath SB: Changes in diapered and nondiapered infant skin over the first month of life. *Pediatr Dermatol* 2000, 17:45-51.
29. Yosipovitch G, Maayan-Metzger A, Merlob P, Sirota L: Skin barrier properties in different body areas in neonates. *Pediatrics* 2000, 106:105-108.
30. Yuan C, Zou Y, Xueqiu Y, Shima K, Miyauchi Y, Naoe A, Naito S, Fujimura T, Hotta M, Kitahara T, Wang X: Properties of Skin in Chinese Infants: Developmental Changes in Ceramides and in Protein Secondary Structure of the Stratum Corneum. *Biomed Res Int* 2017, 2017:3594629.
31. Zhu YH, Song SP, Luo W, Elias PM, Man MQ: Characterization of skin friction coefficient, and relationship to stratum corneum hydration in a normal Chinese population. *Skin Pharmacol Physiol* 2011, 24:81-86.
32. Fluhr JW, Lachmann N, Baudouin C, Msika P, Darlenski R, De Belilovsky C, Bossert J, Colomb E, Burdin B, Haftek M: Development and organization of human stratum corneum after birth: electron microscopy isotropy score and immunocytochemical corneocyte labelling as epidermal maturation's markers in infancy. *Br J Dermatol* 2014, 171:978-986.
33. Kashibuchi N, Hirai Y, O'Goshi K, Tagami H: Three-dimensional analyses of individual corneocytes with atomic force microscope: morphological changes related to age, location and to the pathologic skin conditions. *Skin Res Technol* 2002, 8:203-211.
34. Paus R, Cotsarelis G: The biology of hair follicles. *N Engl J Med* 1999, 341:491-497.
35. Marchini G, Nelson A, Edner J, Lonne-Rahm S, Stavréus-Evers A, Hultenby K: Erythema Toxicum Neonatorum Is an Innate Immune Response to Commensal Microbes Penetrated into the Skin of the Newborn Infant. *Pediatric Research* 2005, 58:613-616.
